# Antimicrobial resistance and documented empirical antibiotic prescribing among cancer patients with culture-confirmed infections at a tertiary referral hospital in Rwanda, 2019–2024

**DOI:** 10.1186/s12879-026-14689-2

**Published:** 2026-10-21

**Authors:** Brandon Niyibizi Asuman, Damas Dukundane, Marrigje Jacoba Kreuger, Simon Ganal, Katarina Stete, Hajo Grundmann, Vanessa M. Eichel

**Affiliations:** 1 Center for Planetary Health, University of Freiburg, Freiburg, Germany; 2 King Faisal Hospital, Kigali, Rwanda; 3 University Medical Center Freiburg, Hugstetterstr. 55, 79106 Freiburg, Germany

**Keywords:** Antimicrobial resistance, Oncology, Cancer, ESKAPEE, Urinary tract infection, Empirical antibiotic therapy, Antimicrobial stewardship, WHO AWaRe, Sub-Saharan Africa, Rwanda

## Abstract

**Background:**

Antimicrobial resistance (AMR) threatens infection management in cancer patients, yet data from sub-Saharan African oncology settings are scarce. We described the microbiology, resistance patterns and documented empirical prescribing among cancer patients with culture-confirmed infection in Rwanda.

**Methods:**

We conducted a retrospective, records-based study of 297 culture-confirmed infection episodes in 286 cancer patients at King Faisal Hospital, Kigali, from 2019 to 2024. Routine susceptibility results were interpreted with the Clinical and Laboratory Standards Institute breakpoints in use at the time. Multidrug resistance (MDR) was classified with the organism-specific definitions of Magiorakos et al., which covered 232 of 254 bacterial isolates. Factors associated with MDR were examined by modified Poisson regression (adjusted prevalence ratios, aPR) with sensitivity analyses. Empirical regimens documented in the electronic medical record (EMR; 105 episodes, 100 inpatient) were compared with the WHO AWaRe framework and the 2022 Rwanda Standard Treatment Guidelines.

**Results:**

Urinary tract infections (UTIs) predominated (172/297; 57.9%). *Escherichia coli* (101/297; 34.0%), *Klebsiella* spp. (32/297; 10.8%) and *Enterococcus* spp. (25/297; 8.4%) were the commonest isolates, and ESKAPEE pathogens accounted for 201/297 episodes (67.7%). MDR was identified in 138 of 232 covered isolates (59.5%; 95% CI 53.1–65.6) and 138 of 214 classifiable isolates (64.5%; 95% CI 57.9–70.6); restricting to more complete panels increased the estimate. Ceftriaxone resistance was high (*E. coli* 65/87, 74.7%; *Klebsiella* spp. 20/29, 69.0%), and carbapenem resistance in *Acinetobacter* spp. was 8/13 (61.5%). MDR prevalence did not differ by infection type (aPR versus UTI: bloodstream 0.94, 95% CI 0.67–1.31; skin and soft tissue 0.98, 95% CI 0.72–1.34) and was lower among gram-positive isolates (aPR 0.42, 95% CI 0.21–0.85), owing to *Enterococcus* spp. Adherence of documented regimens to the national guideline was lowest for UTIs (1/43; 2.3%). Outpatient empirical prescribing was rarely documented in the EMR and could not be evaluated.

**Conclusions:**

MDR was common across all infection types, and ceftriaxone, the most frequently documented empirical agent, showed limited in vitro activity against the prevailing isolates. Whether oncology-specific empirical protocols based on local antibiograms are warranted should be evaluated prospectively, including clinical outcomes, alongside AWaRe-aligned stewardship and better documentation of prescribing.

**Supplementary Information:**

The online version contains supplementary material available at https://doi.org/10.1186/s12879-026-14689-2.

## Plain language summary

Antibiotic resistance is rising worldwide, and cancer patients are particularly vulnerable because their immune system is weakened by both their disease and its treatment. Most studies on this problem come from wealthy countries; very little is known about cancer patients in sub-Saharan Africa. We reviewed the medical records of 286 cancer patients treated at a large hospital in Kigali, Rwanda, who had a laboratory-confirmed infection between 2019 and 2024 (297 infection episodes in total). Urinary tract infections were by far the most common, and the bacteria causing them were often resistant to ceftriaxone, the antibiotic most commonly used as first-line treatment at the hospital. Bacteria of the so-called “ESKAPEE” group, which often carry resistance, accounted for two thirds of all infections, and about three in five of the bacteria that could be assessed were resistant to at least three classes of antibiotics. Among the inpatient cases for whom the initial antibiotic treatment was recorded in the medical record, the choice frequently differed from what national Rwandan guidelines recommend; for outpatient cases, this information was rarely captured in the electronic record at all. Our findings suggest that hospitals treating cancer patients in Rwanda should review which antibiotics are used as first choice in light of the resistance seen in their own patients, assess infection-prevention measures, improve documentation of antibiotic prescribing (particularly for outpatients), and ensure that data from cancer wards feed into the national surveillance system for antibiotic resistance.

**Supplementary Information:**

The online version contains supplementary material available at https://doi.org/10.1186/s12879-026-14689-2.

## Background

Antimicrobial resistance (AMR) is recognised by the World Health Organization and the US Centers for Disease Control and Prevention as one of the foremost threats to global public health [1]. Antibiotic misuse, overuse, and limited diagnostic capacity are key drivers of rising resistance [2], and recent projections suggest that AMR could be associated with around 10 million additional deaths annually by 2050 in the absence of substantial intervention [3].

Bacterial infections are a leading cause of morbidity and mortality among cancer patients [4]. Increased susceptibility reflects immunosuppression from the malignancy itself and from systemic anti-cancer therapy, radiotherapy, surgery, and indwelling devices such as urinary and central venous catheters. Cancer patients face a threefold higher risk of infection-related death compared with patients without cancer [4]. Antibiotics play a critical prophylactic and therapeutic role for patients undergoing surgery [5], stem cell transplantation [6], and systemic anti-cancer therapy- or radiation-induced neutropenia [7]. The spectrum of infections varies with cancer type, neutropenic status, and treatment regimen [8], but gram-negative organisms are consistently reported as predominant pathogens [9]. Bloodstream infections are frequently caused by *Escherichia coli*, *Klebsiella pneumoniae*, and *Pseudomonas aeruginosa*, with rising resistance to commonly used agents [10].

Multidrug-resistant (MDR) pathogens have become increasingly prevalent in oncology settings [11, 12], with implications for prognosis and treatment planning [13]. Resistant ESKAPE/ESKAPEE pathogens (*Enterococcus* spp., *Staphylococcus aureus*, *Klebsiella pneumoniae*, *Acinetobacter* spp., *Pseudomonas aeruginosa*, *Enterobacter* spp., and *Escherichia coli*), methicillin-resistant *S. aureus* (MRSA), carbapenem-resistant Enterobacterales (CRE), and extended-spectrum β-lactamase (ESBL)–producing Enterobacterales feature prominently in this population [14]. A previous study at Kigali University Teaching Hospital reported third-generation cephalosporin resistance of 31.4% in *E. coli* and 58.7% in *Klebsiella* isolates, with 82% of *S. aureus* isolates classified as MRSA [15]. The WHO AWaRe (Access, Watch, Reserve) classification [16] and the 2022 Rwanda Standard Treatment Guidelines [17] provide frameworks for appropriate empirical prescribing, but adherence remains suboptimal across low- and middle-income countries (LMICs) including Rwanda [18].

Despite the heightened vulnerability of cancer patients to drug-resistant infection, oncology-specific empirical treatment guidance is rare in LMIC settings, and the evidence base from sub-Saharan African oncology populations is particularly limited [19, 20]. This gap has direct consequences for practice in Rwanda. National empirical recommendations are derived from general hospital populations, whereas cancer patients differ systematically in immunosuppression, cumulative healthcare exposure, use of indwelling urinary and vascular devices, and prior antibiotic pressure; hospital-wide antibiograms may therefore misrepresent the pathogens and resistance phenotypes that clinicians encounter on the cancer ward, and empirical regimens chosen on that basis may be systematically mismatched to the organisms actually isolated. To our knowledge, no published study has described bacterial profiles, antimicrobial resistance patterns, or the appropriateness of documented empirical antibiotic prescribing among cancer patients in Rwanda.

We therefore aimed to (i) describe the spectrum of culture-confirmed infections and their bacterial aetiology in this population, (ii) quantify antimicrobial resistance and multidrug resistance among bacterial isolates, (iii) identify factors associated with multidrug resistance, and (iv) compare documented empirical prescribing with the WHO AWaRe framework and the 2022 Rwanda Standard Treatment Guidelines. We hypothesised that multidrug resistance would be common in this population, that its prevalence would differ by infection type, and that resistance to third-generation cephalosporins, which are widely used for empirical treatment, would be common among the organisms isolated.

## Methods

### Study design and setting

We conducted a retrospective, records-based observational study of bacterial isolates and antimicrobial resistance among cancer patients with culture-confirmed infections at King Faisal Hospital, Kigali, Rwanda, between January 2019 and December 2024. King Faisal Hospital is a 160-bed tertiary referral and teaching hospital affiliated with Africa Health Sciences University. The hospital is currently expanding to 600 beds and houses one of the three major cancer-care facilities in Rwanda, providing comprehensive oncology services including systemic anti-cancer therapy, surgical oncology, and palliative care [21]. Although case accrual spanned six years, patients were not followed longitudinally and no time-to-event or incidence estimates were derived: each infection episode contributed a single cross-sectional observation of resistance status at the time of culture. The design is therefore a retrospective, records-based study with cross-sectional analysis at the level of the infection episode, and we report it in accordance with the STROBE checklist for cross-sectional studies (Additional file 1). Data abstraction from the electronic medical record was performed between 7 January and 30 June 2025, after the end of the clinical observation period.

### Study population and case definition

Eligible cases were pseudonymised electronic medical records (EMR) of patients with a confirmed malignancy who had at least one culture-confirmed infection during the study period. Records were included if the patient had an active cancer diagnosis at the time of the infection episode, if a clinical specimen yielded growth of a pathogen reported by the hospital laboratory, and if the infection site could be classified from the clinical record. Records were excluded if the malignancy could not be confirmed from the record, if the infection episode fell outside the study period, if the patient was managed exclusively at another facility, or if the microbiological report was incomplete to the extent that neither organism identification nor susceptibility results could be retrieved. Culture-negative episodes lay outside the sampling frame by design and were not an exclusion criterion: the study describes the resistance profile of culture-confirmed infections and cannot characterise culture-negative or untested infections (see Limitations).

The unit of analysis was the infection episode. A total of 297 infection episodes in 286 unique patients were included; 11 patients contributed two episodes each. In nine of these eleven patients the two episodes were separated in time (median interval 4 months) and involved different infection sites or different organisms; in two patients, two sites were sampled on the same day and the episodes are therefore concurrent rather than sequential. A single isolate was retained per episode, so that episode and isolate counts are identical (*n* = 297) and the terms are used interchangeably in the tables; counts are stated at the patient level (*n* = 286) only where explicitly indicated. Infection type was assigned from the clinical diagnosis documented in the EMR at the time the culture was taken, read together with the specimen source: UTI for a documented urinary tract infection with growth from urine; BSI for a positive blood culture with documented bloodstream infection or sepsis; SSTI for documented wound, abscess, surgical-site, or soft tissue infection with growth from pus, tissue, wound fluid, or wound swab; RTI for documented respiratory infection with growth from sputum, tracheal aspirate, or throat swab; and gastrointestinal/other for documented intra-abdominal, peritoneal, or enteric infection with growth from faeces, peritoneal fluid, or other body fluid. Where the documented diagnosis and the specimen source differed, the documented diagnosis determined the category. Symptom-based criteria, quantitative culture counts, and repeat cultures were not consistently recorded and could not be used to separate infection from colonisation or contamination. We therefore defined a conservative set of episodes in which colonisation or contamination could not be reliably excluded: coagulase-negative staphylococci recovered from blood culture (*n* = 3), bacterial growth from sputum or tracheal aspirate (*n* = 16), and bacterial growth from throat or wound swabs (*n* = 4). All MDR analyses were repeated after excluding these 23 episodes, leaving 231 of the 254 bacterial episodes and 215 of the 232 isolates covered by the MDR definitions.

### Microbiological methods

All microbiological procedures were performed prospectively as part of routine clinical care in the King Faisal Hospital laboratory during the study period, in accordance with hospital standard operating procedures; the present study is a secondary analysis of these routinely generated laboratory and clinical data, and no specimens were obtained or processed for research purposes. Blood samples were inoculated into BD BACTEC bottles (Becton-Dickinson, Franklin Lakes, NJ, USA) and incubated at 37 °C in the BACTEC automated system; bottles flagged as positive were subcultured on solid media. Other clinical samples were directly inoculated onto blood agar, MacConkey agar, and Sabouraud-dextrose agar plates and incubated at 37 °C. Plates were examined after 18–24 h; exudate cultures were monitored daily for up to 72 h. Recovered isolates were identified using the BD Phoenix automated microbiology system as the routine standard. When automated panels were unavailable because of supply interruption, or when the automated identification was inconclusive, identification was completed with analytical profile index (API) strips (bioMérieux, Marcy-l’Étoile, France) and conventional biochemical tests: API 20E for Enterobacterales and the API 20 NE system for non-lactose-fermenting gram-negative bacilli; *Staphylococcus* isolates were identified by catalase and coagulase testing with novobiocin susceptibility testing, and *Streptococcus* isolates by catalase testing, haemolysis pattern, and susceptibility testing, as appropriate. Antimicrobial susceptibility testing (AST) followed the same decision rule: the BD Phoenix system was used when panels were available and Kirby–Bauer disc diffusion otherwise. Neither identification nor susceptibility methods were applied selectively by patient group. Susceptibility testing used BD Phoenix or disc diffusion throughout the study period, except for the final weeks of 2024, when the laboratory introduced VITEK 2 (bioMérieux); four bacterial isolates were sampled from December 2024 onwards. The testing method used for each isolate was not recorded. AST results were interpreted by the laboratory according to the Clinical and Laboratory Standards Institute (CLSI) breakpoints in use at the time of testing. The edition applied was not recorded for each isolate, and historical results were not reclassified against updated breakpoints; changes in breakpoints over time may therefore have caused some misclassification of isolates near the breakpoints (see Limitations).

First-generation cephalosporins (cefazolin, cephalexin) were grouped as one variable during data collection. Tetracycline was usually tested as the class representative; doxycycline was tested in a minority of isolates. Methicillin resistance in *S. aureus* was determined by oxacillin. Gentamicin was tested at routine concentration for enterococci; because Magiorakos et al. define MDR using high-level aminoglycoside resistance only, this category was not applied to enterococci. Amoxicillin, cephalexin and polymyxin B, which are not named in the published Enterobacterales table, were assessed within the category of their class, and the *P. aeruginosa* definition was also applied to the four non-*aeruginosa Pseudomonas* isolates.

Phenotypic confirmatory testing for resistance mechanisms — extended-spectrum β-lactamase (ESBL) production, carbapenemase production, and inducible clindamycin resistance (D-test) — was not part of the routine workflow throughout the study period, and no such result was recorded in the laboratory information system for any of the 297 episodes. Resistance mechanisms could therefore not be reported. Carbapenem-resistant Enterobacterales (CRE), carbapenem-resistant non-fermenters, and methicillin-resistant *Staphylococcus aureus* (MRSA) were defined phenotypically from the recorded susceptibility categories, and no molecular characterisation was undertaken. For MRSA we used oxacillin resistance as the defining criterion rather than the organism label recorded by the laboratory, because the MRSA label was not consistently applied to every oxacillin-resistant isolate; all *S. aureus* isolates are therefore reported as a single group (*n* = 11) in Tables 2 and 5 and in Additional file 2, Section S1, with the methicillin-resistant proportion given as a susceptibility result (see Limitations).

### Operational definitions

Multidrug resistance (MDR) was defined according to Magiorakos et al. [22] as acquired non-susceptibility to at least one agent in three or more antimicrobial categories, using the organism-specific category lists of that proposal: Table 1 for *S. aureus*, Table 2 for *Enterococcus* spp., Table 3 for Enterobacterales (Enterobacteriaceae in the source), Table 4 for *P. aeruginosa*, and Table 5 for *Acinetobacter* spp. The categories, agents, and exclusions are listed in Additional file 2, Section S2. The organism-specific criteria were applied to each isolate as follows. (i) Only agents listed for the organism were assessed; agents that reach therapeutic concentrations only in urine (nitrofurantoin, nalidixic acid) did not contribute for Enterobacterales, phenicols and nitrofurans did not contribute for *Enterococcus* spp., for which the high-level aminoglycoside category was not applied because only routine gentamicin was tested, and oxacillin formed its own category for *S. aureus*. (ii) Agents to which the organism is intrinsically resistant according to CLSI M100, as marked in Tables 4 and 5, were not assessed. (iii) A category was counted as tested when at least one of its agents had a recorded result, and as non-susceptible when at least one of its agents was reported resistant.

**Table 1:** Demographic and clinical characteristics of cancer patients with culture-confirmed infection, King Faisal Hospital, Kigali, Rwanda, 2019–2024

Category	Variable	Frequency	Percentage (%)
Treatment setting (*n* = 297)	Inpatient	192	64.6
	Outpatient	105	35.4
Underlying cancer type (*n* = 297)	Solid organ malignancy	262	88.2
	Haematological malignancy	35	11.8
Specific cancer type (*n* = 297)	Prostate	68	22.9
	Colorectal	43	14.5
	Brain and nervous system	24	8.1
	Breast	23	7.7
	Stomach	22	7.4
	Leukaemia	19	6.4
	Head and neck	14	4.7
	Urinary bladder	12	4.0
	Lymphoma	12	4.0
	Ovarian	6	2.0
	Liver	6	2.0
	Other (≤ 1.7% each)	48	16.2
Cancer stage (*n* = 248)	Stage I	6	2.4
	Stage II	39	15.7
	Stage III	71	28.6
	Stage IV	132	53.2

**Table 2:** Distribution of isolated microorganisms by clinical sample type among cancer patients, King Faisal Hospital, Kigali, Rwanda, 2019–2024

Gram type	Organism	Total *N* (%)	Urine *N* (%)	Blood *N* (%)	Pus *N* (%)	Tracheal aspirate *N* (%)	Body fluid *N* (%)	Sputum *N* (%)	Other *N* (%)
Gram-negative rods	*Escherichia coli*	101 (34.0)	81 (80.2)	7 (6.9)	11 (10.9)	–	1 (1.0)	1 (1.0)	–
	*Klebsiella* spp.	32 (10.8)	15 (46.9)	9 (28.1)	3 (9.4)	1 (3.1)	1 (3.1)	1 (3.1)	2 (6.3)
	*Pseudomonas aeruginosa*	19 (6.4)	6 (31.6)	5 (26.3)	4 (21.1)	3 (15.8)	1 (5.3)	–	–
	*Acinetobacter* spp.	13 (4.4)	5 (38.5)	–	–	5 (38.5)	2 (15.4)	1 (7.7)	–
	*Proteus* spp.	10 (3.4)	6 (60.0)	1 (10.0)	2 (20.0)	1 (10.0)	–	–	–
	*Enterobacter* spp.	8 (2.7)	3 (37.5)	3 (37.5)	1 (12.5)	–	1 (12.5)	–	–
	Other gram-negative rodsᵃ	15 (5.1)	8 (53.3)	3 (20.0)	2 (13.3)	–	1 (6.7)	–	1 (6.7)
Gram-positive cocci	*Enterococcus* spp.	25 (8.4)	16 (64.0)	4 (16.0)	2 (8.0)	–	3 (12.0)	–	–
	*Streptococcus* spp.	11 (3.7)	3 (27.3)	2 (18.2)	2 (18.2)	–	–	1 (9.1)	3 (27.3)
	*Staphylococcus aureus* (incl. MRSA)	11 (3.7)	1 (9.1)	2 (18.2)	3 (27.3)	1 (9.1)	2 (18.2)	1 (9.1)	1 (9.1)
	Coagulase-negative staphylococci	9 (3.0)	5 (55.6)	3 (33.3)	1 (11.1)	–	–	–	–
Yeast cells	*Candida albicans*	40 (13.5)	22 (55.0)	5 (12.5)	4 (10.0)	1 (2.5)	2 (5.0)	4 (10.0)	2 (5.0)
	Other yeasts	3 (1.0)	–	–	–	–	–	–	3 (100)
**Total**		**297 (100)**	171	44	35	12	14	9	12
**ESKAPEE pathogensᵇ**		**201 (67.7)**	122 (60.7)	30 (14.9)	24 (11.9)	9 (4.5)	10 (5.0)	4 (2.0)	2 (1.0)

ᵃOther gram-negative rods comprised Providencia spp. (*n* = 4), Pseudomonas spp. (non-aeruginosa, *n* = 4), Serratia spp. (*n* = 2), Morganella morganii (*n* = 2), Salmonella spp. (*n* = 1), Stenotrophomonas maltophilia (*n* = 1), and one unspeciated gram-negative bacillus. ᵇ ESKAPEE pathogens were defined as E. coli (*n* = 101), K. pneumoniae (*n* = 24 of the 32 Klebsiella isolates), P. aeruginosa (*n* = 19), Acinetobacter spp. (*n* = 13), Enterobacter spp. (*n* = 8), Enterococcus spp. (*n* = 25), and S. aureus including MRSA (*n* = 11), giving 201 of 297 episodes (67.7%). The eight non-pneumoniae Klebsiella isolates and the four non-aeruginosa Pseudomonas isolates were not counted as ESKAPEE pathogens. Species-level identification for Acinetobacter was not consistently available

**Table 3:** Factors associated with multidrug resistance (MDR) among cancer patients with culture-confirmed bacterial infection, King Faisal Hospital, Kigali, Rwanda, 2019–2024 (*n* = 214 isolates classifiable under the Magiorakos definitions)

Feature	Category	MDR (*n*=138)	Non-MDR (*n*=76)	Unadj. *p*	aPR (95% CI)	Adj. *p*
Age, median (IQR)	—	60 (46–67)	58.5 (44.8–66.3)	0.847	0.95 (0.86–1.05)	0.357
Sex	Female	50 (36.2%)	34 (44.7%)	0.283	0.86 (0.68–1.08)	0.188
	Male (ref)	88 (63.8%)	42 (55.3%)		Ref.	
Treatment setting	Outpatient	57 (41.3%)	28 (36.8%)	0.622	1.01 (0.79–1.30)	0.913
	Inpatient (ref)	81 (58.7%)	48 (63.2%)		Ref.	
Cancer stage	I	0 (0.0%)	3 (4.9%)	0.013	—ᶜ	
	II	24 (20.3%)	7 (11.5%)		—ᶜ	
	III	27 (22.9%)	22 (36.1%)		—ᶜ	
	IV	67 (56.8%)	29 (47.5%)		—ᶜ	
Infection type	UTI (ref)	86 (62.3%)	41 (53.9%)	0.627	Ref.	
	BSI	19 (13.8%)	14 (18.4%)		0.94 (0.67–1.31)	0.707
	SSTI	20 (14.5%)	14 (18.4%)		0.98 (0.72–1.34)	0.905
	RTI/GI/other	13 (9.4%)	7 (9.2%)		1.00 (0.68–1.47)	0.996
Gram stain	Gram-positive	6 (4.3%)	15 (19.7%)	< 0.001	0.42 (0.21–0.85)	0.015
	Gram-negative (ref)	132 (95.7%)	61 (80.3%)		Ref.	

Continuous variables are presented as median (first–third quartile); categorical variables as frequency (%). Unadj. p = unadjusted p-value (chi-squared test, with Yates’ correction for 2 × 2 tables; Mann–Whitney U test for age). aPR = adjusted prevalence ratio from modified Poisson regression (log link, robust variance; *n* = 214), adjusted simultaneously for age (standardised; per standard deviation of 15.2 years), sex, treatment setting, gram-stain category, and infection type; RTI (*n* = 13) and gastrointestinal/other (*n* = 7) infections were combined because of small numbers. Ref. = reference category. ᶜ Cancer stage was not included in the model (35 missing values); its unadjusted difference rests on three stage I isolates and was not significant in an adjusted complete-case model (joint robust Wald test *p* = 0.26, stages I and II combined, complete-case *n* = 179)

The lower MDR prevalence among gram-positive isolates was not present after exclusion of Enterococcus spp. (aPR 0.94, 95% CI 0.54–1.66). Excluded from this table: 22 isolates of organisms not covered by the Magiorakos definitions and 18 covered isolates with fewer than three categories tested. Sensitivity analyses are reported in Additional file 2, Section S2

**Table 4:** Antimicrobial resistance among gram-negative rods isolated from cancer patients, King Faisal Hospital, Kigali, Rwanda, 2019–2024

Antibiotic agent	*E. coli* (*n* = 101)	*Klebsiella* spp. (*n* = 32)	*P. aeruginosa* (*n* = 19)	*Acinetobacter* spp. (*n* = 13)	*Proteus* spp. (*n* = 10)	*Enterobacter* spp. (*n* = 8)	Other GNR (*n* = 10)	*Providencia* spp. (*n* = 4)	*Salmonella* spp. (*n* = 1)
Ceftriaxone	65/87 (74.7)	20/29 (69.0)	Rᵈ	7/7 (100.0)	4/8 (50.0)	6/7 (85.7)	2/5 (40.0)	3/4 (75.0)	1/1 (100.0)
Cefotaxime	36/52 (69.2)	6/10 (60.0)	Rᵈ	3/3 (100.0)	1/2 (50.0)	4/4 (100.0)	1/5 (20.0)	1/2 (50.0)	1/1 (100.0)
Cefuroxime	59/84 (70.2)	18/27 (66.7)	Rᵈ	Rᵈ	5/9 (55.6)	Rᵈ	5/6 (83.3)	4/4 (100.0)	1/1 (100.0)
Cefepime	33/60 (55.0)	13/23 (56.5)	2/13 (15.4)	5/9 (55.6)	0/5 (0.0)	3/5 (60.0)	3/8 (37.5)	1/3 (33.3)	0/1 (0.0)
Ceftazidime	1/1 (100.0)	1/1 (100.0)	6/15 (40.0)	3/3 (100.0)	1/1 (100.0)	–	2/4 (50.0)	–	–
Ampicillin	65/71 (91.5)	Rᵈ	Rᵈ	Rᵈ	3/5 (60.0)	Rᵈ	4/4 (100.0)	Rᵈ	–
Amoxicillin	27/31 (87.1)	Rᵈ	Rᵈ	Rᵈ	1/1 (100.0)	Rᵈ	2/2 (100.0)	Rᵈ	–
Amoxicillin–clavulanate	41/64 (64.1)	17/26 (65.4)	Rᵈ	Rᵈ	2/7 (28.6)	Rᵈ	3/4 (75.0)	Rᵈ	–
Piperacillin–tazobactam	14/77 (18.2)	9/25 (36.0)	3/14 (21.4)	7/10 (70.0)	0/8 (0.0)	3/7 (42.9)	1/8 (12.5)	3/4 (75.0)	0/1 (0.0)
Aztreonam	24/43 (55.8)	3/9 (33.3)	3/10 (30.0)	Rᵈ	0/2 (0.0)	3/4 (75.0)	1/4 (25.0)	0/1 (0.0)	1/1 (100.0)
Meropenem	1/87 (1.1)	5/29 (17.2)	6/18 (33.3)	8/13 (61.5)	1/8 (12.5)	1/8 (12.5)	0/7 (0.0)	3/4 (75.0)	0/1 (0.0)
Imipenem	1/40 (2.5)	3/17 (17.6)	1/4 (25.0)	7/7 (100.0)	1/3 (33.3)	0/2 (0.0)	0/2 (0.0)	3/3 (100.0)	–
Amikacin	9/86 (10.5)	5/28 (17.9)	6/19 (31.6)	5/13 (38.5)	0/8 (0.0)	1/7 (14.3)	2/9 (22.2)	1/4 (25.0)	0/1 (0.0)
Gentamicin	24/87 (27.6)	11/28 (39.3)	2/13 (15.4)	8/11 (72.7)	1/10 (10.0)	2/8 (25.0)	3/8 (37.5)	3/4 (75.0)	0/1 (0.0)
Ciprofloxacin	30/38 (78.9)	15/19 (78.9)	7/17 (41.2)	9/11 (81.8)	2/4 (50.0)	3/5 (60.0)	3/8 (37.5)	3/3 (100.0)	–
Levofloxacin	45/67 (67.2)	11/16 (68.8)	7/16 (43.8)	6/9 (66.7)	2/5 (40.0)	2/3 (66.7)	1/9 (11.1)	3/4 (75.0)	0/1 (0.0)
Norfloxacin	23/42 (54.8)	2/6 (33.3)	0/1 (0.0)	0/1 (0.0)	0/1 (0.0)	1/1 (100.0)	0/1 (0.0)	–	–
Nitrofurantoin	17/71 (23.9)	8/13 (61.5)	Rᵈ	Rᵈ	Rᵈ	0/3 (0.0)	2/2 (100.0)	Rᵈ	–
TMP/SMX	68/89 (76.4)	18/30 (60.0)	Rᵈ	4/8 (50.0)	6/10 (60.0)	4/7 (57.1)	5/9 (55.6)	3/3 (100.0)	0/1 (0.0)
Colistin	1/52 (1.9)	0/15 (0.0)	0/14 (0.0)	0/10 (0.0)	Rᵈ	0/6 (0.0)	0/7 (0.0)	Rᵈ	0/1 (0.0)
Polymyxin B	2/44 (4.5)	0/11 (0.0)	0/15 (0.0)	0/6 (0.0)	Rᵈ	0/6 (0.0)	1/2 (50.0)	Rᵈ	0/1 (0.0)

Denominators are the number of isolates of each organism for which the agent appears in the recorded antibiogram; they differ between cells because susceptibility panels varied with organism, specimen, and reagent availability. The number of isolates of each organism recovered in the cohort is given in the column headings. ᵈ R indicates intrinsic resistance, for which a susceptibility result is not reported; – indicates that the agent was not tested against any isolate of that organism. Other GNR comprises Pseudomonas spp. (non-aeruginosa, *n* = 4), Serratia spp. (*n* = 2), Morganella morganii (*n* = 2), Stenotrophomonas maltophilia (*n* = 1), and one unspeciated gram-negative bacillus. TMP/SMX, trimethoprim–sulfamethoxazole; GNR, gram-negative rods

Values are the number of resistant isolates over the number of isolates tested against that agent (%)

**Table 5:** Antimicrobial resistance among gram-positive cocci isolated from cancer patients, King Faisal Hospital, Kigali, Rwanda, 2019–2024

Antibiotic agent	*Enterococcus* spp. (*n* = 25)	*S. aureus* (incl. MRSA) (*n* = 11)	*Streptococcus* spp. (*n* = 11)	CoNS (*n* = 9)
Penicillin	5/6 (83.3)	3/3 (100.0)	–	2/2 (100.0)
Ampicillin	4/8 (50.0)	6/6 (100.0)	2/8 (25.0)	6/6 (100.0)
Amoxicillin–clavulanate	1/6 (16.7)	1/2 (50.0)	1/8 (12.5)	1/3 (33.3)
Oxacillin	–	4/10 (40.0)	–	6/7 (85.7)
Cefazolin	Rᵈ	0/1 (0.0)	2/2 (100.0)	1/1 (100.0)
Cefuroxime	Rᵈ	–	1/2 (50.0)	1/1 (100.0)
Cephalexin	Rᵈ	0/3 (0.0)	2/2 (100.0)	1/1 (100.0)
Vancomycin	0/20 (0.0)	0/5 (0.0)	0/11 (0.0)	0/8 (0.0)
Linezolid	0/1 (0.0)	0/3 (0.0)	–	0/3 (0.0)
Gentamicin	Rᵈ	1/5 (20.0)	3/8 (37.5)	1/4 (25.0)
Ciprofloxacin	2/9 (22.2)	3/5 (60.0)	1/5 (20.0)	0/4 (0.0)
Levofloxacin	1/9 (11.1)	0/1 (0.0)	1/2 (50.0)	–
Norfloxacin	4/8 (50.0)	0/1 (0.0)	1/3 (33.3)	1/1 (100.0)
Ofloxacin	3/4 (75.0)	2/3 (66.7)	2/5 (40.0)	1/3 (33.3)
Erythromycin	11/15 (73.3)	4/5 (80.0)	2/5 (40.0)	4/6 (66.7)
Clindamycin	Rᵈ	1/3 (33.3)	1/2 (50.0)	1/1 (100.0)
Doxycycline	9/10 (90.0)	1/1 (100.0)	–	–
Tetracyclines	5/5 (100.0)	1/2 (50.0)	1/2 (50.0)	2/3 (66.7)
Chloramphenicol	2/9 (22.2)	1/4 (25.0)	2/6 (33.3)	1/3 (33.3)
Nitrofurantoin	3/15 (20.0)	0/1 (0.0)	0/2 (0.0)	1/3 (33.3)
TMP/SMX	Rᵈ	7/9 (77.8)	5/7 (71.4)	7/8 (87.5)

Denominators are the number of isolates of each organism for which the agent appears in the recorded antibiogram (see Table 4 footnote). ᵈR indicates intrinsic resistance; – indicates that the agent was not tested against any isolate of that organism. Streptococcus spp. includes one alpha-haemolytic isolate. CoNS, coagulase-negative staphylococci; MRSA, methicillin-resistant Staphylococcus aureus; TMP/SMX, trimethoprim–sulfamethoxazole

Values are the number of resistant isolates over the number of isolates tested against that agent (%)

The laboratory reported results as susceptible or resistant, and no separate intermediate category was recorded (any intermediate result would have been counted as resistant). An agent recorded as both susceptible and resistant for the same isolate (10 isolates) was counted as resistant. Untested agents were treated as missing, never as susceptible. (iv) An isolate was classified as MDR when it was non-susceptible in three or more categories; methicillin-resistant *S. aureus* was classified as MDR, as specified by Magiorakos et al. (v) An isolate that was not MDR and had fewer than three categories tested was classified as not classifiable, because MDR could not be excluded. Organisms outside the five definitions (*Streptococcus* spp., *n* = 11; coagulase-negative staphylococci, *n* = 9; *Stenotrophomonas maltophilia*, *n* = 1; one unspeciated gram-negative bacillus) were reported separately as not covered rather than as non-MDR.

The primary estimate is MDR among the 232 covered isolates, which counts not-classifiable isolates as non-MDR and is therefore conservative; the analysis restricted to classifiable isolates (at least three categories tested) uses 214 isolates as denominator. Further sensitivity analyses defined MRSA by the laboratory label rather than by oxacillin resistance, treated enterococcal gentamicin results as high-level gentamicin, widened the fluoroquinolone category to every fluoroquinolone tested, omitted the automatic MRSA rule, counted agents recorded as both susceptible and resistant as susceptible, excluded episodes in which colonisation or contamination could not be excluded, and excluded episodes sampled from December 2024 onwards, when VITEK 2 was introduced. MDR prevalence was also examined among isolates with progressively more complete panels (at least four, five and six categories tested) (Additional file 2, Section S2).

ESKAPEE pathogens were operationally defined to include *E. coli*, *K. pneumoniae*, *P. aeruginosa*, *Acinetobacter* spp. (species-level identification was not consistently available and *Acinetobacter baumannii* specifically could not be distinguished from related species), *Enterobacter* spp., *Enterococcus* spp., and *S. aureus* (including MRSA).

All resistance analyses were restricted to bacterial isolates (*n* = 254), and MDR analyses to the 232 bacterial isolates covered by the Magiorakos definitions. Fungal isolates recovered from the same cohort (*Candida albicans*, *n* = 40; other yeasts, *n* = 3) are reported in the description of the microbiological profile, because they form part of the culture-confirmed infection burden of this population and because their exclusion would misrepresent the composition of the cohort, but they were excluded from every antibacterial resistance analysis, for which antibacterial susceptibility categories do not apply.

Empirical antibiotic prescribing was defined as the antibiotic regimen initiated before culture and susceptibility results were available. The empirical-therapy variable was extracted from the EMR as documented at the time of care; an empirical regimen was recorded for 105 of 297 episodes (35.4%). EMR capture was markedly asymmetric: among inpatient episodes, 100 of 192 (52.1%) had a documented empirical regimen, whereas only 5 of 105 outpatient episodes (4.8%) did so. Outpatient empirical prescribing was therefore systematically under-captured, although clinical practice strongly suggests that antibiotics were administered at the point of care in most such encounters. All analyses of empirical therapy were accordingly confined to the 105 episodes with documented therapy and are interpreted as reflecting predominantly inpatient prescribing practice.

Documented empirical regimens were assessed against two distinct frameworks. For the WHO framework we report AWaRe category concordance, defined as prescription of a regimen listed as a first- or second-choice option for the corresponding clinical syndrome in the WHO AWaRe Antibiotic Book [16]. Because AWaRe is primarily an antibiotic classification and stewardship framework rather than a site-specific prescribing protocol, we use the term concordance rather than adherence for this framework. Adherence to the 2022 Rwanda Standard Treatment Guidelines [17] was defined as prescription of a regimen recommended for the corresponding infection site. Each infection type was mapped to the corresponding syndrome chapters of the AWaRe Antibiotic Book and to the corresponding syndromes of the national guideline: UTI to lower and upper urinary tract infection; BSI to sepsis in adults and febrile neutropenia; SSTI to skin and soft tissue infections (impetigo, erysipelas and cellulitis; wound-related infections; necrotizing fasciitis); RTI to community-acquired and hospital-acquired pneumonia; and gastrointestinal/other to intra-abdominal infections. Both assessments were made at the level of the infection episode and applied to the whole documented regimen, including all agents and their route of administration (16 of 105 regimens contained more than one agent). A regimen not listed for the syndrome in a framework was coded as non-concordant with that framework. Severity of illness, allergy, renal function, and prior antibiotic exposure were not consistently documented and were not considered; the assessment therefore describes agreement of the documented choice with each framework, not clinical appropriateness for the individual patient. One of the 105 episodes could not be assessed. The coding of every documented regimen, by infection type and route, is provided as a decision matrix in Additional file 2, Section S3.

### Data collection

A structured data collection tool implemented in KoBoToolbox captured demographic, clinical, and microbiological variables, including age, sex, cancer type and stage, treatment setting (inpatient versus outpatient), infection type, sample type, organism identification, and complete antimicrobial susceptibility profiles. Data abstraction was performed from pseudonymised EMR by one trained reviewer (BNA), with independent cross-verification by a second reviewer at three-day intervals during data collection; discrepancies were resolved by discussion and, where needed, adjudicated by the clinical supervisor (MJK). In total, 45 of the 297 episodes (15.2%) were cross-verified, the records being selected at random using a fixed seed so that the subset was not chosen by either abstractor. Across the ten key variables abstracted for each record, 450 fields were compared: 43 of 45 records (95.6%) were identical in every field, and 448 of 450 fields (99.6%) agreed. The two discrepancies concerned cancer stage and sample type; organism identification, infection type, and the recorded resistance status agreed in every record. Both discrepancies were resolved by discussion with reference to the source record. The data collection tool was pilot-tested on 20 records before full implementation. All isolates were processed using identical microbiology protocols and no systematic differences in measurement methods between groups were identified.

### Statistical analysis

Continuous variables were summarised using medians and interquartile ranges (IQR); categorical variables were reported as n/N (%), with the relevant denominator stated throughout. Binomial 95% confidence intervals (CI, Wilson score method) are given for all proportions derived from fewer than 20 tested isolates. Antimicrobial susceptibility patterns were cross-tabulated by bacterial species and are presented as the number of resistant isolates over the number tested, because not every agent was tested against every isolate. Associations between patient characteristics and MDR were examined using chi-squared tests, with Yates’ continuity correction for 2 × 2 tables; the Mann–Whitney U test was used for age. WHO AWaRe category concordance and adherence to the 2022 Rwanda Standard Treatment Guidelines [16, 17] were compared across infection types using chi-squared tests, with Fisher’s exact test (Freeman–Halton extension) reported alongside because several cells contained fewer than five observations; both are shown in Table 6. These comparisons were restricted to the 105 episodes with documented empirical therapy.

**Table 6:** WHO AWaRe category concordance and adherence to the Rwanda Standard Treatment Guidelines by infection type among oncology patients with empirical therapy documented in the electronic medical record, King Faisal Hospital, Kigali, Rwanda, 2019–2024 (*n* = 105; 100/105 [95.2%] inpatient)

Infection type	WHO AWaRe concordance *n*/*N* (%)	*p*-value	National guideline adherence *n*/*N* (%)	*p*-value
Bloodstream infection	22/28 (78.6)	0.529 (0.59)	9/28 (32.1)	0.001** (< 0.001)
Respiratory tract infection	6/8 (75.0)		4/8 (50.0)	
Skin and soft tissue infection	15/23 (65.2)		4/23 (17.4)	
Urinary tract infection	27/43 (62.8)		1/43 (2.3)	
Gastrointestinal/other infection	2/2 (100)		1/2 (50.0)	

Concordance with the WHO AWaRe Antibiotic Book and adherence to the national guideline were defined as prescription of an empirical agent consistent with the recommendation for the identified infection site under each framework (see Methods). p-values are from the chi-squared test across all five infection types, with Fisher’s exact test (Freeman–Halton extension) shown in parentheses because several cells contain fewer than five observations; the double asterisk denotes *p* < 0.01. The analysis is restricted to the 105 of 297 episodes (35.4%) with an empirical regimen documented in the EMR, of which 104 could be assessed against both frameworks (one episode had no guideline assessment recorded) and 100 were inpatient encounters; the estimates therefore reflect predominantly inpatient practice

Factors associated with MDR were examined among the 214 classifiable isolates using modified Poisson regression (log link, robust variance) to estimate adjusted prevalence ratios (aPR) with 95% CI. Prevalence ratios were chosen as the primary measure because MDR was common, so that odds ratios would overstate the corresponding prevalence ratios. The model included standardised age, sex, treatment setting, gram-stain category, and infection type, entered simultaneously, with UTI as the reference category because it was the largest group. Cancer stage was not included in the main model because it was missing for 35 of the 214 classifiable isolates; it was examined in a complete-case model with a joint robust Wald test, combining stages I and II because no stage I isolate was MDR. To limit sparse-data bias, RTI and gastrointestinal/other infections (20 classifiable isolates combined) were merged into one category in the primary model; the five-category model is reported as a sensitivity analysis, and logistic regression with Firth’s penalised likelihood was fitted alongside conventional logistic regression, for both the four- and five-category models, to assess small-sample bias.

Sensitivity analyses of the model comprised: inclusion of all 232 covered isolates with not-classifiable isolates counted as non-MDR; generalised estimating equations (Poisson, exchangeable correlation, patient as cluster) to account for the 11 patients who contributed two episodes; restriction to the first bacterial episode per patient (*n* = 207); exclusion of episodes in which colonisation or contamination could not be excluded (*n* = 197); exclusion of *Enterococcus* spp., whose MDR classification is constrained by panel coverage (*n* = 203); and exclusion of episodes sampled from December 2024 onwards, when VITEK 2 was introduced (*n* = 212).

Because susceptibility methods and CLSI breakpoints may have changed during the study period, the change in MDR prevalence across calendar years was examined by modified Poisson regression on year of sampling. MDR classification and all regression analyses were performed in Python (pandas, statsmodels, SciPy); the code, which reproduces every MDR classification, prevalence estimate, and model reported here from the study dataset, is provided as Additional file 2, Section S4. Descriptive statistics were computed in SPSS version 29 (IBM Corp., Armonk, NY, USA). A two-sided p-value < 0.05 was considered statistically significant.

### Ethics

All ethical aspects were conducted in accordance with the Declaration of Helsinki. Ethical approval was obtained from the Institutional Review Board of King Faisal Hospital (protocol KFH/2025/291/IRB) and the Ethics Committee of the University of Freiburg (reference EK-Freiburg 25−1122-S2). The requirement for written informed consent was waived given the retrospective, pseudonymised nature of the dataset.

## Results

### Demographic and clinical characteristics of the study participants

The study included 297 culture-confirmed infection episodes in 286 unique cancer patients. Because the unit of analysis is the infection episode, the counts below and in Tables 1, 2, 3, 4, 5 and 6 refer to episodes (equivalently, to the single isolate recovered per episode) unless stated otherwise. Of the 297 episodes, 173 (58.2%) occurred in male and 124 (41.8%) in female patients. Among men, the 60–69-year age group was the largest subgroup, whereas women showed a more balanced distribution across age categories (Table 1). The majority of episodes occurred in inpatients (192/297; 64.6%). Solid organ malignancies accounted for 262/297 episodes (88.2%) and haematological malignancies for 35/297 (11.8%). Prostate cancer was the most common underlying diagnosis (68/297; 22.9%), followed by colorectal cancer (43/297; 14.5%), reflecting the cancer epidemiology of Rwanda, where prostate cancer is the leading malignancy in men [23]. Among the 248 episodes with recorded staging, stage IV disease accounted for the largest share (132/248; 53.2%), indicating that many patients had advanced metastatic cancer at the time of infection.

### Infection types and their bacterial aetiology

**Fig. 1:**
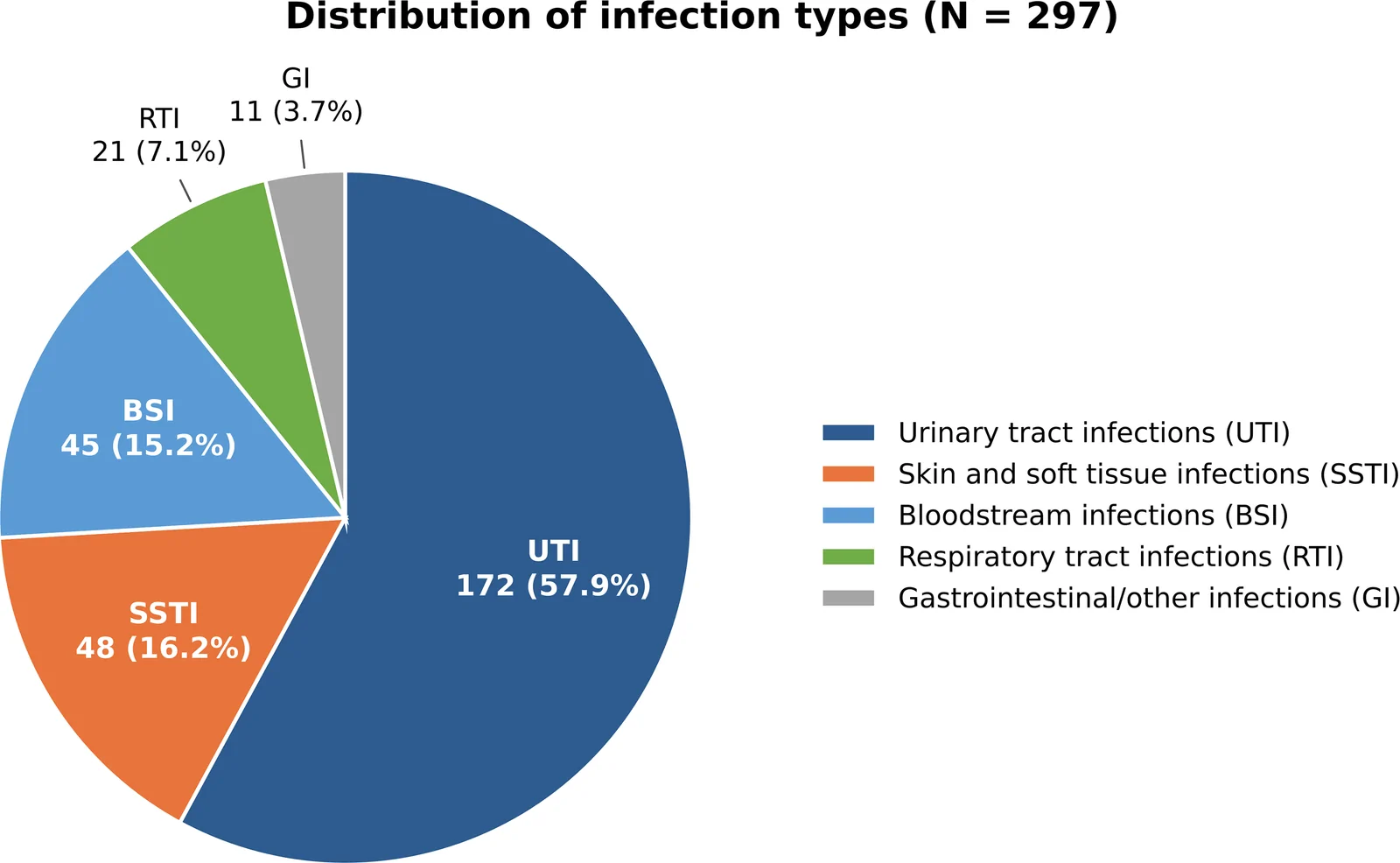
Distribution of infection types among cancer patients with culture-confirmed infections at King Faisal Hospital, Kigali, Rwanda, 2019–2024 (*n* = 297)

Urinary tract infections were the most common infection type (172/297; 57.9%), followed by skin and soft tissue infections (48/297; 16.2%) and bloodstream infections (45/297; 15.2%) (Fig. 1; Table 2).

Gram-negative bacilli dominated the microbiological profile (198/297 episodes; 66.7%), followed by gram-positive cocci (56/297; 18.9%); fungal isolates accounted for the remainder (*Candida albicans* 40/297, 13.5%; other yeasts 3/297, 1.0%) and were excluded from all subsequent resistance analyses. Of the 254 bacterial isolates, 198 (78.0%) were gram-negative and 56 (22.0%) gram-positive. *Escherichia coli* was the single most common pathogen (101/297 episodes, 34.0%; 101/254 bacterial isolates, 39.8%), with most isolates (81/101; 80.2%) recovered from urine. *Klebsiella* spp. (combining *K. pneumoniae* and other *Klebsiella* spp.) were the second most common gram-negative group (32/297; 10.8%). Among gram-positive organisms, *Enterococcus* spp. were most prevalent (25/297; 8.4%) and were also predominantly recovered from urine (16/25; 64.0%). ESKAPEE pathogens (as defined in Methods) accounted for 201 of 297 episodes (67.7%) and for 201 of 254 bacterial isolates (79.1%), of which 122 (60.7%) were recovered from urine specimens.

The distribution of organisms by clinical specimen type is shown in Table 2, and the bacterial aetiology of each infection syndrome is given in Additional file 2, Section S1. Specimen type and infection site did not correspond one-to-one (48 episodes were classified as SSTI, whereas 36 isolates were recovered from pus). Among the 150 bacterial UTI episodes, *E. coli* predominated (82/150; 54.7%), followed by *Enterococcus* spp. (15/150; 10.0%) and *K. pneumoniae* (10/150; 6.7%). Bloodstream infections (*n* = 40) were caused most often by *K. pneumoniae* (9/40; 22.5%), *E. coli* (7/40; 17.5%), and *P. aeruginosa* (5/40; 12.5%). Skin and soft tissue infections (*n* = 40) were dominated by *E. coli* (12/40; 30.0%), whereas respiratory tract infections (*n* = 16) were dominated by *Acinetobacter* spp. (6/16; 37.5%) and *P. aeruginosa* (3/16; 18.8%).

### Antimicrobial resistance and multidrug resistance patterns

Under the organism-specific definitions, 138 of the 232 covered isolates were MDR (59.5%; 95% CI 53.1–65.6), as were 138 of the 214 classifiable isolates (64.5%; 95% CI 57.9–70.6). By organism group, MDR was identified in 114 of 160 Enterobacterales (71.2%), 10 of 13 *Acinetobacter* spp. (76.9%), 8 of 23 *Pseudomonas* spp. (34.8%), 6 of 11 *S. aureus* (54.5%), and none of 25 *Enterococcus* spp., 14 of which could not be classified. Across the alternative specifications and exclusions, MDR prevalence ranged from 58.6% to 60.1% of covered isolates. Restricting the analysis to isolates with more complete panels increased the estimate (67.8% with at least four and 74.9% with at least six categories tested), so the primary estimate is conservative (Additional file 2, Section S2). Resistance among WHO priority pathogens was substantial (see Additional file 2, Section S5 for the full breakdown by priority category). Within the WHO critical-priority group, third-generation cephalosporin resistance was identified in 99 of 142 tested Enterobacterales (69.7%; 95% CI 62–77) and carbapenem resistance in 12 of 148 tested Enterobacterales (8.1%; 95% CI 4.7–13.6); carbapenem resistance reached 8 of 13 tested *Acinetobacter* spp. isolates (61.5%; 95% CI 36–82) and 7 of 22 tested *Pseudomonas* spp. isolates (31.8%; 95% CI 16–53; *P. aeruginosa* and non-*aeruginosa* species pooled). Among high-priority pathogens, MRSA — defined phenotypically by oxacillin resistance — was identified in 4 of 10 tested *S. aureus* isolates (40.0%; 95% CI 17–69), and no vancomycin-resistant enterococci were detected (0/20 tested; 95% CI 0–16). Estimates derived from fewer than 20 tested isolates are imprecise and should be regarded as preliminary.

Among gram-negative bacilli (Table 4), ceftriaxone resistance was high in every Enterobacterales group tested: *E. coli* 65/87 (74.7%), *Klebsiella* spp. 20/29 (69.0%), *Enterobacter* spp. 6/7 (85.7%; 95% CI 49–97), and *Providencia* spp. 3/4 (75.0%; 95% CI 30–95); all seven tested *Acinetobacter* isolates were ceftriaxone-resistant. Trimethoprim–sulfamethoxazole resistance was similarly high, particularly in *E. coli* (68/89; 76.4%). Fluoroquinolone resistance was substantial across the Enterobacterales (*E. coli* ciprofloxacin 30/38, 78.9%; levofloxacin 45/67, 67.2%). Carbapenem resistance was concentrated in the non-fermenters and in *Providencia* spp.: meropenem resistance reached 8/13 (61.5%) in *Acinetobacter* spp. and 6/18 (33.3%) in *P. aeruginosa*, whereas it remained rare in *E. coli* (1/87; 1.1%). Piperacillin–tazobactam retained the best in vitro activity among the β-lactams tested against *E. coli* (14/77 resistant; 18.2%), as did amikacin (9/86; 10.5%) among the aminoglycosides, and few tested gram-negative isolates were reported resistant to colistin or polymyxin B.

Among gram-positive cocci (Table 5), no vancomycin or linezolid resistance was detected in any tested isolate. Trimethoprim–sulfamethoxazole resistance was high in *S. aureus* (7/9; 77.8%), *Streptococcus* spp. (5/7; 71.4%), and coagulase-negative staphylococci (7/8; 87.5%). Erythromycin resistance affected 11 of 15 tested *Enterococcus* isolates (73.3%) and 4 of 5 tested *S. aureus* isolates (80.0%). Oxacillin resistance was more frequent among coagulase-negative staphylococci (6/7; 85.7%) than among *S. aureus* (4/10; 40.0%). Chloramphenicol showed moderate activity, with resistance in 2 of 9 tested *Enterococcus* isolates (22.2%) and 1 of 4 tested *S. aureus* isolates (25.0%).

### Factors associated with multidrug resistance

Among the 214 classifiable isolates (Table 3), MDR prevalence did not differ in unadjusted analyses by age, sex, treatment setting, or infection type (*p* = 0.627). Cancer stage showed an unadjusted difference (*p* = 0.013) that rested on three stage I isolates and showed no ordered pattern among stages II–IV, stage III having the lowest proportion; it was not significant in an adjusted complete-case model with stages I and II combined (joint robust Wald *p* = 0.26, *n* = 179). Gram-positive isolates were less often MDR than gram-negative isolates (6/21 versus 132/193; *p* < 0.001).

In the primary modified Poisson model, infection type was not associated with MDR: compared with UTI, the adjusted prevalence ratio was 0.94 (95% CI 0.67–1.31) for bloodstream infection, 0.98 (0.72–1.34) for skin and soft tissue infection, and 1.00 (0.68–1.47) for respiratory and gastrointestinal/other infection combined. Age, sex, and treatment setting were likewise not associated with MDR. Gram-positive isolates had a lower adjusted MDR prevalence (aPR 0.42, 95% CI 0.21–0.85; *p* = 0.015); this difference was not present when *Enterococcus* spp. were excluded (aPR 0.94, 95% CI 0.54–1.66), indicating that it is driven by the enterococcal isolates, whose classification is constrained by the limited panel. The absence of an association between infection type and MDR was consistent across every sensitivity analysis: the five-category model, inclusion of all covered isolates, generalised estimating equations, restriction to first episodes, exclusion of possible colonisation or contamination, exclusion of episodes from the VITEK 2 period, and conventional and Firth-penalised logistic regression (four- and five-category models), whose estimates were nearly identical, indicating no material sparse-data bias (Additional file 2, Section S2). There was no evidence of a linear trend in MDR prevalence over the study period (prevalence ratio per calendar year 1.02, 95% CI 0.95–1.09; *p* = 0.63).

### Empirical treatment, AWaRe concordance, and guideline adherence

**Fig. 2:**
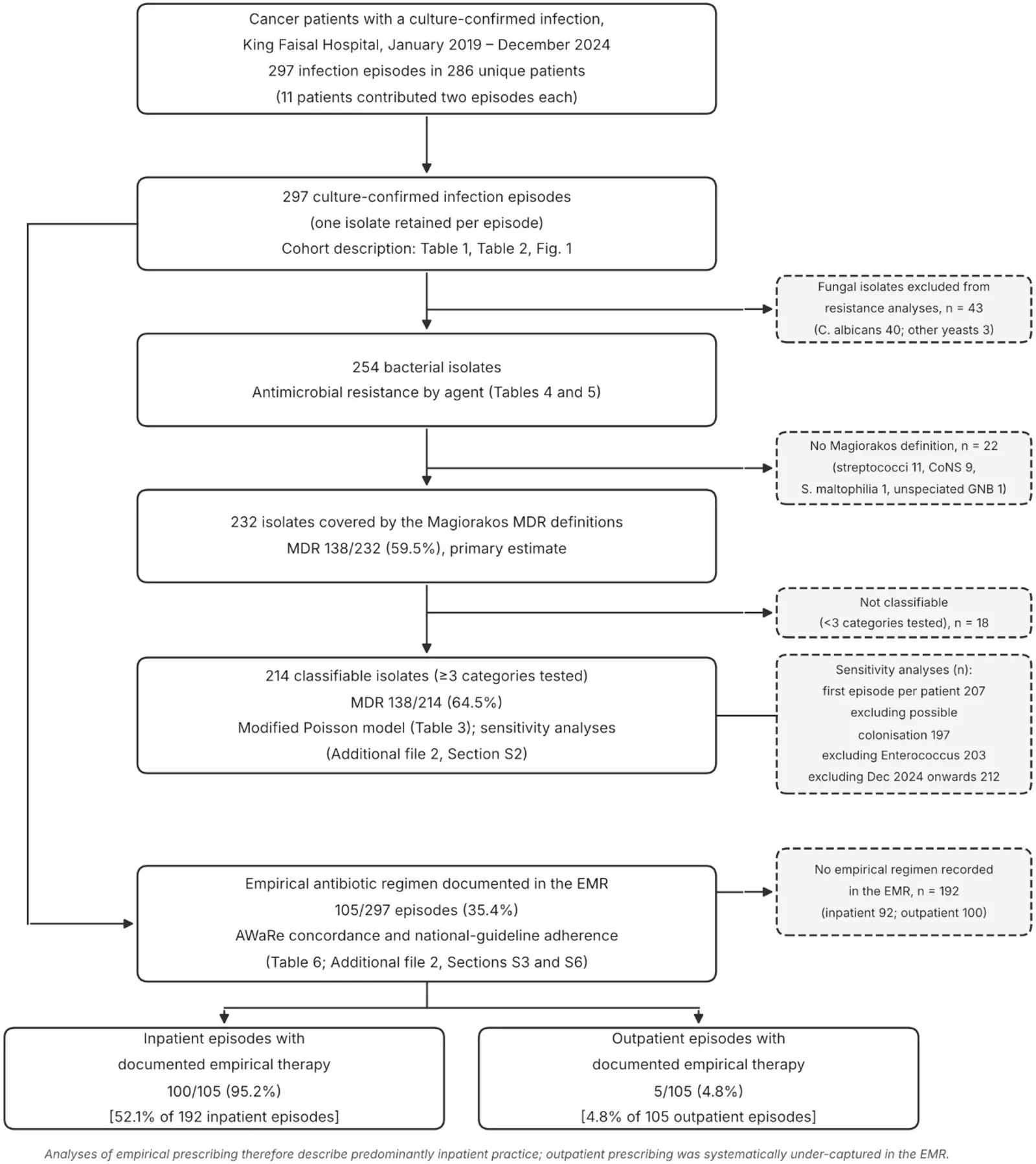
Flow of infection episodes through the study, showing the denominators used in each analysis: 297 culture-confirmed infection episodes; 254 bacterial isolates included in resistance analyses; 232 isolates covered by the MDR definitions, of which 214 were classifiable and used in the regression analyses; and 105 episodes with empirical therapy documented in the electronic medical record, of which 100 were inpatient encounters

The analyses in this section are restricted to the 105 of 297 episodes (35.4%) for which an empirical antibiotic regimen was documented in the EMR, of which 100 (95.2%) were inpatient encounters. They therefore describe predominantly inpatient prescribing and cannot be generalised either to the cohort as a whole or to outpatient practice (Fig. 2).

A documented empirical antibiotic regimen was available in the EMR for 105 of 297 episodes (35.4%). EMR capture was markedly asymmetric: 100 of 192 inpatient episodes (52.1%) had a documented regimen, compared with only 5 of 105 outpatient episodes (4.8%); outpatient empirical therapy was therefore systematically under-captured, although clinical practice suggests antibiotics were administered in most such encounters. A total of 19 different antibiotic agents were prescribed empirically in the 105 documented episodes; the complete distribution by WHO AWaRe category is provided in Additional file 2, Section S6. Watch-group antibiotics predominated; ceftriaxone was the most frequently prescribed agent (54/105; 51.4%), followed by meropenem (14/105; 13.3%) and ciprofloxacin (6/105; 5.7%). Access-group antibiotics accounted for a notable proportion, with metronidazole (12/105; 11.4%) the most commonly prescribed, followed by amoxicillin–clavulanate (8/105; 7.6%). Use of Reserve-group agents was minimal (polymyxin B, 1/105; 1.0%).

Within this predominantly inpatient subset, WHO AWaRe category concordance and adherence to the 2022 Rwanda Standard Treatment Guidelines were assessed as defined in Methods; both frameworks could be applied to 104 of the 105 documented episodes. The two frameworks differ substantially: whereas the WHO AWaRe framework accommodates Watch-category agents such as ceftriaxone for several common syndromes, the Rwanda national guidelines specify narrower first-line choices. AWaRe category concordance ranged from 27/43 (62.8%) for UTIs to 22/28 (78.6%) for BSIs (2/2 for gastrointestinal/other) and did not differ significantly across infection types (chi-squared *p* = 0.529; Fisher’s exact *p* = 0.59; Table 6). Adherence to national guidelines was substantially lower across all infection types: 9/28 (32.1%) for BSIs, 4/8 (50.0%) for RTIs, 4/23 (17.4%) for SSTIs, 1/43 (2.3%) for UTIs, and 1/2 for gastrointestinal/other; the association between infection type and national-guideline adherence was statistically significant (chi-squared *p* = 0.001; Fisher’s exact *p* < 0.001). All five outpatient episodes with documented therapy were UTIs. Laboratory turnaround time from sampling to microbiology result had a median of 2 days (IQR 2–4; *n* = 293), and 82% of results were available within 4 days. Four episodes were excluded from this calculation: one had no recorded result date, and three had intervals that are not clinically plausible (two negative, of−59 and−30 days, and one of 153 days) and most probably reflect date-entry errors in the EMR; retaining them left the median and interquartile range unchanged.

## Discussion

This retrospective study of 297 culture-confirmed infection episodes among cancer patients at a tertiary referral hospital in Rwanda describes a substantial burden of antimicrobial resistance with potential clinical implications. UTIs were the predominant infection diagnosis (172/297; 57.9%), *E. coli* was the single most common pathogen (34.0%), and ESKAPEE organisms comprised 67.7% of all episodes and 79.1% of bacterial isolates. Applying organism-specific definitions, close to three in five covered bacterial isolates were multidrug-resistant (59.5%; 64.5% of classifiable isolates), and MDR prevalence was similar across infection types, so that bloodstream, skin and soft tissue, and urinary isolates carried a comparable burden. Ceftriaxone resistance was high across the Enterobacterales (*E. coli* 74.7%, *Enterobacter* spp. 85.7%, *Klebsiella* spp. 69.0% of tested isolates), and carbapenem resistance in *Acinetobacter* spp. reached 61.5% in a small subset of isolates. Documented empirical prescribing frequently diverged from national guidance, particularly for UTIs (1 of 43 regimens adherent).

The predominance of gram-negative organisms (66.7%) is consistent with previous reports from Rwanda and with oncology-based series in other LMICs and high-income settings, in which rising ESBL- and carbapenem-resistant phenotypes are increasingly reported among immunocompromised hosts. The large share of ESKAPEE pathogens points to an elevated risk of difficult-to-treat infections in oncology populations. Third-generation cephalosporin resistance in *E. coli* reached 74.7% of tested isolates in our cohort, more than twice the 31.4% reported by Ntirenganya and colleagues at Kigali University Teaching Hospital in 2015 [15], while the *Klebsiella* spp. figure (69.0%) also exceeded the 58.7% reported a decade earlier. Recent hospital-wide King Faisal Hospital laboratory data from 2024 to 2025 (unpublished) indicate *E. coli* ceftriaxone resistance of approximately 40–45%, which is well below the level we observed in the oncology population over the same period; the gap between our cohort and the contemporaneous hospital-wide figure suggests that the difference from the 2015 data reflects the oncology case mix at least as much as a uniform secular rise in resistance, and the two explanations cannot be separated without a common sampling frame. Sutherland and colleagues likewise described widespread resistance in a Rwandan referral hospital in 2019 [24], and resistance in urinary and bloodstream isolates has been described at other Rwandan hospitals [25, 26]. The high ceftriaxone resistance we observed is also consistent with regional studies from Uganda, Ethiopia, and Zimbabwe [27–29] and raises questions about the use of third-generation cephalosporins as first-line empirical therapy in settings with high ESBL prevalence [20, 30]. Ceftriaxone was the agent most often documented for empirical treatment in this cohort; the resistance rates observed here therefore support evaluating whether it remains an appropriate empirical choice in the oncology population studied, and whether oncology-specific adaptation of the national framework is necessary.

Comparisons with these earlier reports must, however, take case mix into account. The Rwandan studies cited above sampled all clinical isolates from general tertiary hospital populations rather than an oncology cohort, and differed correspondingly in the distribution of infection types, specimen sources, prior healthcare exposure, and antibiotic pressure. Because urinary isolates were over-represented in our cohort relative to a general medical population, part of the difference in resistance rates is likely attributable to case mix rather than to a secular increase in resistance, and the two explanations cannot be separated in the absence of a common sampling frame.

The substantial carbapenem non-susceptibility observed in *Acinetobacter* spp. (8/13 tested isolates; 61.5%) raises therapeutic and infection-control concerns given the limited effective alternatives and the toxicity and costs of available last-resort drugs such as polymyxins. The small denominator (*n* = 13) limits the precision of this estimate, which carries a wide 95% CI (36–82%), and the finding should be regarded as preliminary and confirmed in larger samples, ideally through coordinated multi-centre surveillance.

Two features of the MDR estimates deserve emphasis. First, MDR classification from routine antibiograms depends on which categories were tested: isolates tested against few categories cannot reach the MDR threshold, so counting them as non-MDR understates prevalence. This is most visible for *Enterococcus* spp., none of which was classified as MDR although the routine panel covered a median of only two Magiorakos categories, and it explains the lower MDR prevalence among gram-positive isolates; for this reason we report not-classifiable isolates separately and present estimates restricted to isolates with more complete panels alongside the conservative primary estimate. Second, the proportion of isolates resistant to at least one tested agent (218/254; 85.8%) is a much broader measure than MDR and should not be compared with MDR estimates from other studies. Unlike a recent multi-country African analysis in which inpatient status was associated with resistance [31], we found no association between treatment setting and MDR, although outpatients in our cohort were patients with active cancer attending a tertiary oncology service and not representative of community infection.

### Clinical and public health implications

#### Empirical antibiotic protocols warrant review and prospective evaluation

Ceftriaxone was the most frequently documented empirical agent in our cohort (54/105; 51.4%), yet showed limited in vitro activity against the prevailing gram-negative isolates (*E. coli* 74.7%, *Klebsiella* spp. 69.0%, and *Enterobacter* spp. 85.7% of tested isolates resistant). This suggests that third-generation cephalosporins may provide inadequate in vitro coverage for hospital-managed oncology infection in this setting, and that empirical regimens could be re-examined on the basis of locally stratified antibiograms that account for treatment setting, recent antibiotic exposure, and catheterisation status. Carbapenem-sparing alternatives such as piperacillin–tazobactam (*E. coli* resistance 14/77, 18.2%) and amikacin (9/86; 10.5%) merit formal evaluation within locally adapted protocols rather than immediate adoption as first-line therapy. Our data derive from culture-positive episodes and do not include clinical outcomes, illness severity, prior antibiotic exposure, or catheter status, and in vitro susceptibility does not reliably predict clinical cure: pharmacokinetics, tissue penetration, source control, and host immune status also determine outcome. Any change to empirical policy should therefore be accompanied by prospective evaluation of clinical endpoints, including mortality, length of stay, and delays to cancer treatment.

#### Antimicrobial stewardship could be aligned with WHO AWaRe principles

The high reliance on Watch-group agents and the low concordance with national guidance among documented regimens, particularly for UTIs (1 of 43 documented UTI regimens; 2.3%), identify plausible targets for stewardship intervention: prescribing audit with feedback, clinical decision support integrated in the EMR, formulary restriction of key broad-spectrum agents, and prescriber education tailored to oncology contexts. Because these observations rest on the subset of episodes with documented therapy, the magnitude of non-adherence in routine practice — especially in outpatients — remains uncertain and should be quantified prospectively before intervention targets are set.

#### UTI management pathways in oncology merit review

Given the preponderance of UTIs in our cohort, which accounted for most MDR isolates in absolute terms (86/138), targeted measures merit evaluation. These include minimisation of unnecessary urinary catheter use, aseptic insertion and maintenance technique, pre-treatment urine culture, and susceptibility-guided intravenous-to-oral step-down, all of which could reduce both selection pressure and clinical morbidity.

#### The pathogen mix indicates priorities for infection prevention and control

We did not assess infection prevention and control (IPC) structures, practices, or compliance at the study site, and therefore cannot evaluate existing IPC performance or attribute the observed resistance to specific IPC gaps. The pathogen distribution nevertheless indicates which organisms transmission-based measures would need to address: ESKAPEE organisms accounted for 67.7% of episodes, and carbapenem non-susceptibility was concentrated in *Acinetobacter* spp., an organism with well-documented environmental persistence. These findings provide a rationale for a dedicated IPC assessment in the oncology unit, covering hand hygiene, cleaning of high-touch surfaces, and catheter and central-line care bundles, and for determining on that basis whether pathogen-specific (vertical) measures such as admission screening and contact precautions are warranted in this population.

#### Electronic medical record capture of empirical prescribing should be improved

Empirical regimens were recorded in the EMR for only 4.8% of outpatient encounters in this cohort, compared with 52.1% of inpatient encounters. This gap represents a major blind spot for stewardship and surveillance. Standardised structured fields for empirical antibiotic indication, agent, dose, and duration should be introduced for both inpatient and outpatient encounters, ideally with prompts triggered at the point of prescribing. Reliable EMR capture is a prerequisite for any meaningful audit of community-level prescribing practice in this population.

#### Microbiology capacity and surveillance integration could be strengthened

Consistent susceptibility testing with internal and external quality assurance, timely reporting, and integration of oncology-unit isolate data into national AMR surveillance could enable more responsive, evidence-based updates to national prescribing guidance, and will help close the persistent LMIC oncology data gap.

### Limitations

Several limitations should be considered when interpreting these findings. First, the retrospective, single-centre design and reliance on culture-positive cases drawn from electronic medical records may underrepresent culture-negative infections and may not always distinguish true infection from asymptomatic colonisation, particularly for urine and respiratory samples. Differentiation in this study relied on documented clinical features in combination with positive culture; standardised symptom-based criteria were not consistently available in the source records. In a conservative sensitivity analysis excluding coagulase-negative staphylococci from blood, respiratory specimens, and superficial swabs (23 episodes), MDR prevalence and its associations were materially unchanged, although estimates for the small RTI/GI/other group became less precise; colonisation of the urinary tract, the most common source, could not be addressed in the same way because colony counts and symptoms were not consistently recorded.

Second, restricting the analysis to culture-positive episodes introduces selection bias in a specific direction: cultures are more likely to be obtained from patients with severe, complicated, or treatment-refractory infection, from inpatients, and from those with better access to diagnostic services. The resistance proportions reported here are therefore likely to overestimate the prevalence of resistance in the broader oncology population, and they cannot be used to estimate the incidence of bacterial infection or of AMR, nor to identify patient-level risk factors for acquiring a resistant rather than a susceptible infection.

Third, documentation of empirical antibiotic therapy in the EMR was incomplete and markedly asymmetric: an empirical regimen was recorded for 52.1% of inpatient episodes (100/192) but for only 4.8% of outpatient episodes (5/105), despite a strong clinical likelihood that antibiotics were administered at the point of care. The adherence analyses therefore reflect predominantly inpatient prescribing practice, and the true patterns of outpatient empirical therapy could not be evaluated; improved structured EMR capture for both settings is a prerequisite for any thorough future stewardship assessment. Other fields showed comparable data-quality limitations: three episodes carried sampling and result dates that cannot be reconciled, the MRSA label was applied inconsistently, and neither prior outpatient antibiotic exposure nor indwelling-device duration was reliably recorded. Fourth, we did not collect clinical outcomes. The study cannot therefore establish whether discordant empirical therapy translated into higher mortality, longer hospital stay, delayed systemic anti-cancer therapy, or worse microbiological response, and every statement about the clinical consequences of the resistance observed here is a hypothesis for prospective testing rather than a demonstrated effect.

Fifth, resistance mechanisms were not characterised: phenotypic confirmation of ESBL or carbapenemase production and testing for inducible clindamycin resistance were not routinely available, and no molecular typing was performed, so that CRE and MRSA were defined phenotypically and the genetic basis of the resistance observed remains unknown. Sixth, susceptibility panels were not uniform: the agents tested varied with organism, specimen, and reagent availability, so the denominators in Tables 4 and 5 differ between cells and some agent–organism combinations rest on very few isolates. Because clinicians may request susceptibility testing for additional agents when first-line therapy fails, resistance proportions computed among tested isolates may be modestly upward-biased for the less frequently tested agents. Seventh, susceptibility categories were used as reported by the laboratory, which applied the CLSI breakpoints in use at the time of testing; the edition was not recorded for each isolate, and results were not reclassified against updated breakpoints; changes in breakpoint definitions over the six-year study period, particularly for fluoroquinolones, polymyxins, piperacillin–tazobactam and aminoglycosides, could have introduced temporal variation in resistance categorisation. The laboratory also introduced VITEK 2 in December 2024. MDR prevalence showed no trend across calendar years (prevalence ratio per year 1.02, 95% CI 0.95–1.09), and excluding episodes sampled from December 2024 onwards did not change the estimates (Additional file 2, Section S2), which argues against a large effect of methodological change on the main estimates, although effects on individual agents cannot be excluded.

Eighth, MDR classification from routine antibiograms is constrained by panel composition: 18 covered isolates had fewer than three categories tested and could not be classified, 22 isolates belonged to organisms for which no standard MDR definition exists, enterococcal gentamicin was tested only at routine concentration so that the high-level aminoglycoside category could not be assessed, and the data-collection entries “Cephalosporin I” (cefazolin or cephalexin) and “Tetracyclines” (tetracycline) were assigned to the first-generation cephalosporin and tetracycline categories. Because untested categories can hide resistance, MDR prevalence rose with panel completeness (Additional file 2, Section S2), and the primary estimate should be read as conservative. MDR was derived directly from the recorded antibiograms with the published organism-specific algorithm rather than from a summary resistance variable, and the code is provided (Additional file 2, Section S4).

Ninth, eleven patients contributed two infection episodes each; generalised estimating equations and a first-episode-only analysis both confirmed the stability of the main findings, but residual within-patient clustering cannot be entirely excluded. Finally, the study describes a single tertiary referral centre with a specialised oncology service and comparatively well-developed microbiological capacity; the findings should not be extrapolated to primary or secondary care in Rwanda, or to other LMIC settings, without local validation.

Prospective, multi-centre cohort studies enrolling consecutive oncology admissions at presentation, regardless of subsequent infection status, are required to quantify the true incidence of resistant infection and to identify patient-level and treatment-related risk factors. Molecular epidemiological investigations into resistance mechanisms (ESBL genotypes, carbapenemases, plasmid-mediated transfer) would further inform targeted infection prevention and containment strategies. Despite these limitations, our study provides a contemporary snapshot of AMR in an oncology population in Rwanda through December 2024 and offers concrete data for empirical prescribing and stewardship in this setting.

## Conclusions

In this cohort of Rwandan cancer patients with culture-confirmed infection, multidrug resistance was common across all infection types, and the microbiological profile was dominated by ESKAPEE organisms. Ceftriaxone, the most frequently documented empirical agent, showed limited in vitro activity against the prevailing isolates.

These findings support evaluation of whether oncology-specific empirical protocols based on locally stratified antibiograms are warranted, including careful assessment of alternatives to ceftriaxone, together with strengthened culture-before-treatment practice, improved structured EMR capture of empirical prescribing for both inpatients and outpatients, and integration of oncology-unit AMR data into the Rwandan national AMR surveillance system.

Because the analysis was restricted to culture-positive episodes and did not assess clinical outcomes or infection prevention and control practice, these implications require prospective evaluation before empirical protocols are changed.

## Supplementary Information

Below is the link to the electronic supplementary material.

Supplementary Material 1

## Data Availability

No datasets were generated or analysed during the current study.

## Abbreviations

AMR: Antimicrobial resistance
aPR: Adjusted prevalence ratio
AST: Antimicrobial susceptibility testing
AWaRe: Access, Watch, Reserve
BSI: Bloodstream infection
CI: Confidence interval
CLSI: Clinical and Laboratory Standards Institute
CRE: Carbapenem-resistant Enterobacterales
EMR: Electronic medical records
ESBL: Extended-spectrum β-lactamase
ESKAPEE: *Enterococcus, Staphylococcus aureus, Klebsiella pneumoniae, Acinetobacter* spp., *Pseudomonas aeruginosa, Enterobacter* spp., *Escherichia coli*
GEE: Generalised estimating equations
GI: Gastrointestinal
IQR: Interquartile range
IRB: Institutional Review Board
KFH: King Faisal Hospital
LMIC: Low- and middle-income country
MDR: Multidrug resistance/resistant
MRSA: Methicillin-resistant *Staphylococcus aureus*
PR: Prevalence ratio
RTI: Respiratory tract infection
SSTI: Skin and soft tissue infection
STROBE: Strengthening the Reporting of Observational Studies in Epidemiology
TMP/SMX: Trimethoprim–sulfamethoxazole
UTI: Urinary tract infection
WHO: World Health Organization
