# Supplementary material for: Antimicrobial resistance and documented empirical antibiotic prescribing among cancer patients with culture-confirmed infections at a tertiary referral hospital in Rwanda, 2019–2024
Source: BMC Infect Dis. 2026 Oct 21;26:1865. doi: 10.1186/s12879-026-14689-2 (PMC13689241; doi:10.1186/s12879-026-14689-2)
Supplement: Supplementary file 1 [file 12879_2026_14689_MOESM1_ESM.docx]

**Additional file 2**

Supplementary material for: Antimicrobial Resistance and Documented Empirical Antibiotic Prescribing Among Cancer Patients with Culture-Confirmed Infections at a Tertiary Referral Hospital in Rwanda, 2019–2024

**Contents**

S1. Bacterial aetiology by infection type

S2. Organism-specific MDR classification, MDR by organism group and sensitivity analyses

S3. Decision matrix for WHO AWaRe concordance and national-guideline adherence

S4. Analysis code

S5. Antimicrobial resistance among WHO priority pathogens

S6. Empirical antibiotic agents by WHO AWaRe category

# **S1. Bacterial aetiology by infection type**

**Table S1. Bacterial aetiology by infection type among cancer patients with culture-confirmed infection, King Faisal Hospital, Kigali, Rwanda, 2019–2024 (n = 254 bacterial isolates)**

| **Gram category** | **Organism** | **UTI** | **BSI** | **SSTI** | **RTI** | **GI/other** | **Total** |
| --- | --- | --- | --- | --- | --- | --- | --- |
| Gram-negative rods | *Escherichia coli* | 82 | 7 | 12 | 0 | 0 | 101 |
| Gram-negative rods | *Klebsiella pneumoniae* | 10 | 9 | 4 | 1 | 0 | 24 |
| Gram-negative rods | *Pseudomonas aeruginosa* | 6 | 5 | 4 | 3 | 1 | 19 |
| Gram-negative rods | *Acinetobacter* spp. | 5 | 0 | 1 | 6 | 1 | 13 |
| Gram-negative rods | *Proteus* spp. | 6 | 1 | 2 | 1 | 0 | 10 |
| Gram-negative rods | *Enterobacter* spp. | 3 | 3 | 1 | 0 | 1 | 8 |
| Gram-negative rods | *Klebsiella* spp. (other) | 5 | 0 | 1 | 1 | 1 | 8 |
| Gram-negative rods | *Providencia* spp. | 4 | 0 | 0 | 0 | 0 | 4 |
| Gram-negative rods | *Pseudomonas* spp. (non-*aeruginosa*) | 1 | 2 | 1 | 0 | 0 | 4 |
| Gram-negative rods | *Morganella morganii* | 2 | 0 | 0 | 0 | 0 | 2 |
| Gram-negative rods | *Serratia* spp. | 0 | 0 | 1 | 0 | 1 | 2 |
| Gram-negative rods | *Salmonella* spp. | 0 | 0 | 0 | 0 | 1 | 1 |
| Gram-negative rods | Unspeciated gram-negative bacillus | 1 | 0 | 0 | 0 | 0 | 1 |
| Gram-negative rods | *Stenotrophomonas maltophilia* | 0 | 1 | 0 | 0 | 0 | 1 |
| Gram-positive cocci | *Enterococcus* spp. | 15 | 5 | 4 | 0 | 1 | 25 |
| Gram-positive cocci | *Streptococcus* spp. | 4 | 0 | 3 | 3 | 0 | 10 |
| Gram-positive cocci | Coagulase-negative staphylococci | 5 | 3 | 1 | 0 | 0 | 9 |
| Gram-positive cocci | *Staphylococcus aureus* (incl. MRSA) | 1 | 3 | 5 | 1 | 1 | 11 |
| Gram-positive cocci | *Streptococcus* spp. (alpha-haemolytic) | 0 | 1 | 0 | 0 | 0 | 1 |
| **Total** |  | **150** | **40** | **40** | **16** | **8** | **254** |

Values are numbers of infection episodes (one isolate retained per episode). Restricted to the 254 bacterial isolates; the 43 fungal isolates (*Candida albicans* n = 40, other yeasts n = 3) are excluded. Column totals give the bacterial episodes by infection type (UTI 150, BSI 40, SSTI 40, RTI 16, GI/other 8). UTI, urinary tract infection; BSI, bloodstream infection; SSTI, skin and soft tissue infection; RTI, respiratory tract infection. *Staphylococcus aureus* is reported as a single group because the laboratory MRSA label was not applied consistently; methicillin resistance (oxacillin, 4/10 tested) is given in Section S5.

# **S2. Organism-specific MDR classification, MDR by organism group and sensitivity analyses**

**Table S2.1. MDR classification algorithm**

| **Step** | **Rule** |
| --- | --- |
| 1 | Organism groups and categories as in Magiorakos et al. 2012, Tables 1–5 (Table 4 also applied to the four non-*aeruginosa* *Pseudomonas* isolates). Drugs not listed for a group are not assessed, except same-class agents marked in Table S2.2 (amoxicillin, cephalexin, polymyxin B). |
| 2 | Drugs to which the organism is intrinsically resistant (CLSI M100) are not assessed. |
| 3 | Category tested: ≥1 drug tested. Category non-susceptible: ≥1 drug resistant. Results were recorded as susceptible or resistant; no separate intermediate category was recorded, and any intermediate result would have been counted as resistant. A drug recorded as both susceptible and resistant for the same isolate (10 isolates) was counted as resistant. Untested drugs treated as missing, never as susceptible. |
| 4 | MDR: non-susceptible in ≥3 categories. *S. aureus* resistant to oxacillin (MRSA) classified as MDR (Magiorakos, Table 1). |
| 5 | Not classifiable: not MDR and <3 categories tested. |
| 6 | Not covered: organisms without a Magiorakos definition (streptococci, CoNS, *S. maltophilia*, unspeciated GNB); reported separately. |

Magiorakos AP et al. Clin Microbiol Infect 2012;18:268–81.

**Table S2.2. Antimicrobial categories and agents by organism group**

| **Organism group** | **Category** | **Agents (Magiorakos)** | **Agents tested in this study** |
| --- | --- | --- | --- |
| *S. aureus* | Aminoglycosides | gentamicin | gentamicin |
| *S. aureus* | Anti-staphylococcal beta-lactams | cefoxitin, oxacillin | oxacillin |
| *S. aureus* | Fluoroquinolones | ciprofloxacin, moxifloxacin | ciprofloxacin |
| *S. aureus* | Folate pathway inhibitors | tmp-smx | tmp-smx |
| *S. aureus* | Glycopeptides | teicoplanin, vancomycin | vancomycin |
| *S. aureus* | Lincosamides | clindamycin | clindamycin |
| *S. aureus* | Macrolides | erythromycin | erythromycin |
| *S. aureus* | Oxazolidinones | linezolid | linezolid |
| *S. aureus* | Phenicols | chloramphenicol | chloramphenicol |
| *S. aureus* | Tetracyclines | doxycycline, minocycline, tetracycline | doxycycline, tetracycline |
| *S. aureus* | Ansamycins | rifampicin | not tested |
| *S. aureus* | Fucidanes | fusidic acid | not tested |
| *S. aureus* | Glycylcyclines | tigecycline | not tested |
| *S. aureus* | Lipopeptides | daptomycin | not tested |
| *S. aureus* | Phosphonic acids | fosfomycin | not tested |
| *S. aureus* | Streptogramins | quinupristin-dalfopristin | not tested |
| *S. aureus* | Anti-MRSA cephalosporins | ceftaroline | not tested |
| *Enterococcus* spp. | Aminoglycosides (high-level gentamicin) | gentamicin | not tested (gentamicin tested at routine concentration only; category not applied) |
| *Enterococcus* spp. | Carbapenems | doripenem, imipenem, meropenem | imipenem, meropenem |
| *Enterococcus* spp. | Fluoroquinolones | ciprofloxacin, levofloxacin, moxifloxacin | ciprofloxacin, levofloxacin |
| *Enterococcus* spp. | Glycopeptides | teicoplanin, vancomycin | vancomycin |
| *Enterococcus* spp. | Oxazolidinones | linezolid | linezolid |
| *Enterococcus* spp. | Penicillins | ampicillin | ampicillin |
| *Enterococcus* spp. | Tetracyclines | doxycycline, minocycline, tetracycline | doxycycline, tetracycline |
| *Enterococcus* spp. | Streptomycin (high-level) | streptomycin | not tested |
| *Enterococcus* spp. | Glycylcyclines | tigecycline | not tested |
| *Enterococcus* spp. | Lipopeptides | daptomycin | not tested |
| *Enterococcus* spp. | Streptogramins | quinupristin-dalfopristin | not tested |
| Enterobacterales | Aminoglycosides | amikacin, gentamicin, netilmicin, tobramycin | amikacin, gentamicin |
| Enterobacterales | Antipseudomonal penicillins + BLI | piperacillin-tazobactam, ticarcillin-clavulanate | piperacillin-tazobactam, ticarcillin-clavulanate |
| Enterobacterales | Carbapenems | doripenem, ertapenem, imipenem, meropenem | ertapenem, imipenem, meropenem |
| Enterobacterales | Non-extended-spectrum cephalosporins | cefazolin, cefuroxime | cefazolin, cefuroxime; cephalexin and 'cephalosporin i' (same class, added) |
| Enterobacterales | Extended-spectrum cephalosporins | cefepime, cefotaxime, ceftazidime, ceftriaxone | cefepime, cefotaxime, ceftazidime, ceftriaxone |
| Enterobacterales | Cephamycins | cefotetan, cefoxitin | not tested |
| Enterobacterales | Fluoroquinolones | ciprofloxacin | ciprofloxacin |
| Enterobacterales | Folate pathway inhibitors | tmp-smx | tmp-smx |
| Enterobacterales | Monobactams | aztreonam | aztreonam |
| Enterobacterales | Penicillins | ampicillin | ampicillin; amoxicillin (same class, added) |
| Enterobacterales | Penicillins + BLI | amoxicillin-clavulanate, ampicillin-sulbactam | amoxicillin-clavulanate, ampicillin-sulbactam |
| Enterobacterales | Phenicols | chloramphenicol | chloramphenicol |
| Enterobacterales | Polymyxins | colistin | colistin; polymyxin b (same class, added) |
| Enterobacterales | Tetracyclines | doxycycline, minocycline, tetracycline | doxycycline, tetracycline |
| Enterobacterales | Glycylcyclines | tigecycline | not tested |
| Enterobacterales | Phosphonic acids | fosfomycin | not tested |
| Enterobacterales | Anti-MRSA cephalosporins | ceftaroline | not tested |
| *Pseudomonas* spp. | Aminoglycosides | amikacin, gentamicin, netilmicin, tobramycin | amikacin, gentamicin |
| *Pseudomonas* spp. | Antipseudomonal carbapenems | doripenem, imipenem, meropenem | imipenem, meropenem |
| *Pseudomonas* spp. | Antipseudomonal cephalosporins | cefepime, ceftazidime | cefepime, ceftazidime |
| *Pseudomonas* spp. | Antipseudomonal fluoroquinolones | ciprofloxacin, levofloxacin | ciprofloxacin, levofloxacin |
| *Pseudomonas* spp. | Antipseudomonal penicillins + BLI | piperacillin-tazobactam, ticarcillin-clavulanate | piperacillin-tazobactam, ticarcillin-clavulanate |
| *Pseudomonas* spp. | Monobactams | aztreonam | aztreonam |
| *Pseudomonas* spp. | Polymyxins | colistin, polymyxin b | colistin, polymyxin b |
| *Pseudomonas* spp. | Phosphonic acids | fosfomycin | not tested |
| *Acinetobacter* spp. | Aminoglycosides | amikacin, gentamicin, netilmicin, tobramycin | amikacin, gentamicin |
| *Acinetobacter* spp. | Antipseudomonal carbapenems | doripenem, imipenem, meropenem | imipenem, meropenem |
| *Acinetobacter* spp. | Antipseudomonal fluoroquinolones | ciprofloxacin, levofloxacin | ciprofloxacin, levofloxacin |
| *Acinetobacter* spp. | Antipseudomonal penicillins + BLI | piperacillin-tazobactam, ticarcillin-clavulanate | piperacillin-tazobactam, ticarcillin-clavulanate |
| *Acinetobacter* spp. | Extended-spectrum cephalosporins | cefepime, cefotaxime, ceftazidime, ceftriaxone | cefepime, cefotaxime, ceftazidime, ceftriaxone |
| *Acinetobacter* spp. | Folate pathway inhibitors | tmp-smx | tmp-smx |
| *Acinetobacter* spp. | Penicillins + BLI | ampicillin-sulbactam | ampicillin-sulbactam |
| *Acinetobacter* spp. | Polymyxins | colistin, polymyxin b | colistin, polymyxin b |
| *Acinetobacter* spp. | Tetracyclines | doxycycline, minocycline, tetracycline | doxycycline, tetracycline |

'Cephalosporin I' is the data-collection label for first-generation cephalosporins (cefazolin, cephalexin). 'Tetracyclines' denotes tetracycline. Agents marked 'same class, added' are not named in the published table and were assessed within the category of their class. *Pseudomonas* spp.: Table 4 (*P. aeruginosa*) was also applied to the four non-*aeruginosa* isolates.

**Table S2.3. Intrinsic resistance excluded from assessment**

| **Organism** | **Intrinsic resistance (not assessed)** |
| --- | --- |
| *Klebsiella* | amoxicillin, ampicillin, ticarcillin |
| *Enterobacter* | amoxicillin, amoxicillin-clavulanate, ampicillin, ampicillin-sulbactam, cefazolin, cefoxitin, cefuroxime, cephalexin, cephalosporin i |
| *Proteus* | colistin, doxycycline, nitrofurantoin, polymyxin b, tetracycline, tigecycline |
| *Providencia* | amoxicillin, amoxicillin-clavulanate, ampicillin, cefazolin, cephalexin, cephalosporin i, colistin, doxycycline, nitrofurantoin, polymyxin b, tetracycline, tigecycline |
| *Morganella* | amoxicillin, amoxicillin-clavulanate, ampicillin, cefazolin, cefuroxime, cephalexin, cephalosporin i, colistin, doxycycline, nitrofurantoin, polymyxin b, tetracycline, tigecycline |
| *Serratia* | amoxicillin, amoxicillin-clavulanate, ampicillin, cefazolin, cefoxitin, cefuroxime, cephalexin, cephalosporin i, colistin, nitrofurantoin, polymyxin b |
| *Acinetobacter* | amoxicillin, ampicillin, aztreonam |

Enterococcal gentamicin was tested at routine concentration, as confirmed by the laboratory; the high-level aminoglycoside category was therefore not applied. Reading it as high-level is shown as a sensitivity analysis in Table S2.5.

**Table S2.4. MDR by organism group (n = 232 covered isolates)**

| **Organism group** | **MDR** | **Non-MDR** | **Not classifiable** | **MDR %** | **Median categories tested** |
| --- | --- | --- | --- | --- | --- |
| *Acinetobacter* spp. | 10 | 3 | 0 | 76.9 | 6 |
| Enterobacterales | 114 | 43 | 3 | 71.2 | 8 |
| *Enterococcus* spp. | 0 | 11 | 14 | 0.0 | 2 |
| *Pseudomonas* spp. | 8 | 15 | 0 | 34.8 | 6 |
| *S. aureus* | 6 | 4 | 1 | 54.5 | 5 |

22 isolates not covered by a Magiorakos definition: *Streptococcus* spp. 11 (including 1 alpha-haemolytic), CoNS 9, *S. maltophilia* 1, unspeciated GNB 1.

**Table S2.5. Sensitivity analyses of MDR prevalence**

| **Analysis** | **MDR** | **Covered** | **Classifiable** | **% covered** | **% classifiable** |
| --- | --- | --- | --- | --- | --- |
| Primary (MRSA by oxacillin resistance; routine enterococcal gentamicin not counted) | 138 | 232 | 214 | 59.5 | 64.5 |
| MRSA defined by laboratory label | 137 | 232 | 213 | 59.1 | 64.3 |
| Enterococcal gentamicin read as high-level | 138 | 232 | 217 | 59.5 | 63.6 |
| Fluoroquinolone category widened | 138 | 232 | 218 | 59.5 | 63.3 |
| No automatic MRSA rule | 137 | 232 | 213 | 59.1 | 64.3 |
| Agents recorded as both S and R counted as susceptible | 137 | 232 | 214 | 59.1 | 64.0 |
| Excluding possible colonisation/contamination | 126 | 215 | 197 | 58.6 | 64.0 |
| Excluding episodes sampled from December 2024 onwards (VITEK 2 period) | 137 | 228 | 212 | 60.1 | 64.6 |

Colonisation/contamination exclusion: CoNS in blood (3), sputum/tracheal aspirate (16), throat/wound swab (4).

VITEK 2 period: 4 covered isolates sampled from December 2024 onwards.

Percentages: MDR divided by covered isolates, and MDR divided by classifiable isolates.

**Table S2.6. MDR among covered isolates by completeness of the susceptibility panel**

| **Categories tested** | **MDR** | **Isolates** | **MDR %** | **95% CI** |
| --- | --- | --- | --- | --- |
| ≥3 | 137 | 213 | 64.3 | 57.7–70.4 |
| ≥4 | 137 | 202 | 67.8 | 61.1–73.9 |
| ≥5 | 137 | 197 | 69.5 | 62.8–75.5 |
| ≥6 | 131 | 175 | 74.9 | 67.9–80.7 |

MDR prevalence rises as the panel becomes more complete, because untested categories can hide resistance; the primary estimate is therefore conservative.

**Table S2.7. Adjusted prevalence ratios for MDR across models (reference: UTI, gram-negative)**

| **Model** | **BSI** | **SSTI** | **RTI/GI/other** | **Gram-positive** | **RTI** | **GI/other** |
| --- | --- | --- | --- | --- | --- | --- |
| Primary (Poisson, classifiable) (n = 214) | 0.94 (0.67–1.31) p=0.707 | 0.98 (0.72–1.34) p=0.905 | 1.00 (0.68–1.47) p=0.996 | 0.42 (0.21–0.85) p=0.015 |  |  |
| Five infection groups (n = 214) | 0.94 (0.68–1.32) p=0.739 | 0.99 (0.72–1.35) p=0.940 |  | 0.43 (0.21–0.86) p=0.017 | 1.18 (0.82–1.70) p=0.367 | 0.67 (0.26–1.72) p=0.407 |
| All covered (unclassifiable = non-MDR) (n = 232) | 1.02 (0.72–1.44) p=0.908 | 1.07 (0.78–1.46) p=0.663 | 1.07 (0.71–1.61) p=0.746 | 0.24 (0.12–0.51) p<0.001 |  |  |
| GEE by patient (n = 214) | 0.94 (0.68–1.31) p=0.729 | 1.00 (0.74–1.37) p=0.981 | 0.99 (0.67–1.46) p=0.946 | 0.42 (0.21–0.84) p=0.014 |  |  |
| First episode per patient (n = 207) | 0.92 (0.64–1.32) p=0.650 | 0.97 (0.70–1.34) p=0.847 | 0.99 (0.65–1.49) p=0.948 | 0.43 (0.22–0.87) p=0.018 |  |  |
| Excluding possible colonisation (n = 197) | 0.92 (0.65–1.31) p=0.650 | 1.07 (0.79–1.44) p=0.672 | 0.68 (0.27–1.75) p=0.426 | 0.32 (0.13–0.79) p=0.014 |  |  |
| Excluding *Enterococcus* (n = 203) | 0.89 (0.65–1.23) p=0.489 | 0.93 (0.68–1.26) p=0.638 | 0.91 (0.64–1.31) p=0.628 | 0.94 (0.54–1.66) p=0.840 |  |  |
| Excluding VITEK 2 period (from December 2024) (n = 212) | 0.98 (0.70–1.36) p=0.885 | 0.99 (0.72–1.35) p=0.938 | 1.00 (0.68–1.48) p=0.984 | 0.42 (0.21–0.84) p=0.014 |  |  |
| Logistic (aOR) (n = 214) | 0.85 (0.34–2.08) p=0.716 | 0.94 (0.39–2.28) p=0.889 | 1.01 (0.34–2.94) p=0.991 | 0.19 (0.07–0.52) p=0.001 |  |  |
| Firth logistic (aOR) (n = 214) | 0.84 (0.34–2.06) p=0.705 | 0.93 (0.38–2.24) p=0.867 | 0.98 (0.34–2.83) p=0.969 | 0.21 (0.08–0.57) p=0.002 |  |  |
| Logistic, five infection groups (aOR) (n = 214) | 0.86 (0.35–2.12) p=0.745 | 0.96 (0.39–2.32) p=0.920 |  | 0.19 (0.07–0.53) p=0.001 | 1.90 (0.45–8.00) p=0.379 | 0.39 (0.08–1.95) p=0.253 |
| Firth logistic, five infection groups (aOR) (n = 214) | 0.86 (0.35–2.10) p=0.734 | 0.94 (0.39–2.28) p=0.897 |  | 0.21 (0.08–0.58) p=0.003 | 1.71 (0.42–6.87) p=0.453 | 0.41 (0.08–2.03) p=0.277 |

Adjusted for age, sex, treatment setting, gram stain and infection type. Logistic and Firth rows: adjusted odds ratios; Firth confidence intervals are Wald intervals. Values are ratio (95% CI) p.

Calendar year (unadjusted modified Poisson model on year of sampling): PR per year 1.02 (0.95–1.09) p=0.63. Cancer stage, complete-case analysis (n = 179; stages I and II combined): joint robust Wald test p=0.26.

# **S3. Decision matrix for WHO AWaRe concordance and national-guideline adherence**

**Table S3. Coding of every documented empirical regimen by infection type and route (n = 105 episodes)**

| **Infection** | **Regimen (agents and route as documented)** | **AWaRe categories** | **Combination** | **WHO AWaRe concordant (as coded)** | **National guideline adherent (as coded)** | **Episodes** |
| --- | --- | --- | --- | --- | --- | --- |
| BSI | ceftriaxone IV | Watch | No | Yes | No | 10 |
| BSI | erythromycin oral | Watch | No | No | No | 3 |
| BSI | ceftriaxone IV + levofloxacin oral | Watch + Watch | Yes | Yes | No | 2 |
| BSI | meropenem IV | Watch | No | Yes | Yes | 2 |
| BSI | azithromycin oral + ceftriaxone IV | Watch + Watch | Yes | Yes | No | 1 |
| BSI | cefixime oral + metronidazole oral + doxycycline oral | Watch + Access + Access | Yes | No | Yes | 1 |
| BSI | ceftriaxone IV + metronidazole IV | Watch + Access | Yes | Yes | No | 1 |
| BSI | ceftriaxone IV + vancomycin IV + metronidazole IV | Watch + Watch + Access | Yes | Yes | Yes | 1 |
| BSI | cefuroxime IV | Watch | No | No | No | 1 |
| BSI | meropenem IV + erythromycin oral + metronidazole IV | Watch + Watch + Access | Yes | Yes | Yes | 1 |
| BSI | meropenem IV + vancomycin IV + metronidazole (route not recorded) | Watch + Watch + Access | Yes | Yes | Yes | 1 |
| BSI | piperacillin IV | Watch | No | Yes | Yes | 1 |
| BSI | piperacillin-tazobactam (route not recorded) | Watch | No | Yes | Yes | 1 |
| BSI | tmp-smx oral | Access | No | No | No | 1 |
| BSI | vancomycin IV | Watch | No | Yes | Yes | 1 |
| GI/other | ceftriaxone IV | Watch | No | Yes | Yes | 1 |
| GI/other | meropenem IV | Watch | No | Yes | No | 1 |
| GI/other | tmp-smx oral | Access | No | Not assessed | Not assessed | 1 |
| RTI | ceftriaxone IV | Watch | No | Yes | No | 3 |
| RTI | meropenem IV | Watch | No | Yes | Yes | 2 |
| RTI | amoxicillin-clavulanate IV | Access | No | No | Yes | 1 |
| RTI | metronidazole IV | Access | No | No | No | 1 |
| RTI | metronidazole IV + vancomycin IV | Access + Watch | Yes | Yes | Yes | 1 |
| SSTI | ceftriaxone IV | Watch | No | Yes | No | 10 |
| SSTI | meropenem IV | Watch | No | No | No | 3 |
| SSTI | amoxicillin-clavulanate IV | Access | No | No | Yes | 2 |
| SSTI | ciprofloxacin oral | Watch | No | No | No | 2 |
| SSTI | cloxacillin oral | Access | No | Yes | Yes | 2 |
| SSTI | ampicillin IV + metronidazole IV | Access + Access | Yes | No | No | 1 |
| SSTI | ceftriaxone IV + metronidazole IV | Watch + Access | Yes | Yes | No | 1 |
| SSTI | flucloxacillin IV | Access | No | Yes | No | 1 |
| SSTI | meropenem IV + vancomycin IV | Watch + Watch | Yes | Yes | No | 1 |
| UTI | ceftriaxone IV | Watch | No | Yes | No | 21 |
| UTI | amoxicillin-clavulanate IV | Access | No | No | No | 3 |
| UTI | ciprofloxacin oral | Watch | No | Yes | No | 3 |
| UTI | meropenem IV | Watch | No | No | No | 3 |
| UTI | ceftriaxone IV + metronidazole IV | Watch + Access | Yes | Yes | No | 2 |
| UTI | amoxicillin-clavulanate IV + ciprofloxacin IV | Access + Watch | Yes | No | No | 1 |
| UTI | amoxicillin-clavulanate oral | Access | No | No | No | 1 |
| UTI | ceftriaxone IV + cloxacillin IV | Watch + Access | Yes | Yes | Yes | 1 |
| UTI | cefuroxime oral | Watch | No | No | No | 1 |
| UTI | levofloxacin IV | Watch | No | No | No | 1 |
| UTI | levofloxacin oral | Watch | No | No | No | 1 |
| UTI | metronidazole IV | Access | No | No | No | 1 |
| UTI | piperacillin-tazobactam (route not recorded) | Watch | No | No | No | 1 |
| UTI | polymyxin b IV | Reserve | No | No | No | 1 |
| UTI | tmp-smx oral | Access | No | No | No | 1 |
| UTI | vancomycin IV | Watch | No | No | No | 1 |
| **Total** |  |  |  |  |  | **105** |

Assessment unit: the whole documented regimen. Concordance = regimen listed as first- or second-choice for the syndrome in the WHO AWaRe Antibiotic Book; adherence = regimen recommended for the infection site in the 2022 Rwanda Standard Treatment Guidelines. One episode (GI/other, tmp-smx) was not assessed. Oral and intravenous use of the same agent are shown separately because route was considered when classifying the regimen. AWaRe category per agent: current WHO AWaRe classification.

Syndrome mapping (WHO AWaRe Antibiotic Book chapters): UTI, lower and upper urinary tract infection; BSI, sepsis in adults and febrile neutropenia; SSTI, impetigo, erysipelas and cellulitis, wound-related infections and necrotizing fasciitis; RTI, community-acquired and hospital-acquired pneumonia; GI/other, intra-abdominal infections.

# **S4. Analysis code**

The Python code below reproduces, from the study dataset, every MDR classification, prevalence estimate, sensitivity analysis and regression model reported in the manuscript, Table 3 and Section S2. To run it, save the text below as a Python file (for example, analysis_code.py) and run: python analysis_code.py <dataset.xlsx> [output_folder].

```
"""
Additional file 2, Section S4. Analysis code.

Antimicrobial resistance and documented empirical antibiotic prescribing among
cancer patients with culture-confirmed infections, King Faisal Hospital, Kigali,
Rwanda, 2019-2024.

Reproduces, from the study dataset, every multidrug-resistance (MDR)
classification, prevalence estimate, sensitivity analysis and regression model
reported in the manuscript, Table 3 and Additional file 2 (Section S2).

Usage
    python analysis_code.py <dataset.xlsx> [output_folder]

Requirements
    Python 3.9 or later, pandas, numpy, scipy, statsmodels, patsy, openpyxl
    (tested with pandas 3.0, numpy 2.5, scipy 1.18, statsmodels 0.15)

MDR classification follows Magiorakos et al., Clin Microbiol Infect 2012;18:268-81,
Tables 1 to 5. The category lists below contain only agents tested in the
study laboratory; categories for which no agent was tested are listed in
Additional file 2 (Section S2). Agents not named in the published tables but belonging to
the same class (amoxicillin, cephalexin, polymyxin B) are assessed within that
category. Non-aeruginosa Pseudomonas isolates are classified with Table 4.
An agent recorded as both susceptible and resistant for the same isolate is
counted as resistant (a sensitivity analysis counts it as susceptible).

The dataset columns 'Is bacterial' and 'First bacterial episode per patient'
were derived during data collection and are used as recorded.
"""
import sys
import os
import json
from math import sqrt

import numpy as np
import pandas as pd
import patsy
from scipy import stats
import statsmodels.api as sm
import statsmodels.formula.api as smf

DATA = sys.argv[1] if len(sys.argv) > 1 else 'AMR_ONC_study_dataset_annotated.xlsx'
OUT = sys.argv[2] if len(sys.argv) > 2 else 'results'
os.makedirs(OUT, exist_ok=True)

ORG = 'Microbiological Findings/Types of Isolated Organisms'
GRAM = 'Microbiological Findings/Gram Stain Result_2'
SUS_COLS = ['Antibiotic Results/Gram Positive/Susceptibility',
            'Antibiogram Results/Gram Negative/Susceptibility']
RES_COLS = ['Antibiotic Results/Gram Positive/Resistance',
            'Antibiogram Results/Gram Negative/Resistance']

# ----------------------------------------------------------------------------
# 1. Agent names as recorded in the dataset -> standard names
#    "Cephalosporin I" is the data-collection grouping of first-generation
#    cephalosporins (cefazolin, cephalexin); "Tetracyclines" is tetracycline.
# ----------------------------------------------------------------------------
ALIASES = {
    'tmp-smx (co-trimoxazole)': 'tmp-smx', 'tmp/smx (co-trimoxazole)': 'tmp-smx',
    'polymixin b': 'polymyxin b',
    'amoxicillin + clavulanate': 'amoxicillin-clavulanate',
    'piperacillin + tazobactam': 'piperacillin-tazobactam',
    'ticarcillin + clavulanic acid': 'ticarcillin-clavulanate',
    'ampicillin + sulbactam': 'ampicillin-sulbactam',
    'tetracyclines': 'tetracycline',
    'penicillin g': 'penicillin',
}


def agent(name):
    n = name.strip().lower()
    return ALIASES.get(n, n)


def agents(row, cols):
    out = set()
    for c in cols:
        v = row[c]
        if pd.isna(v):
            continue
        out |= {agent(t) for t in str(v).split(',') if t.strip()}
    return out


# ----------------------------------------------------------------------------
# 2. Antimicrobial categories by organism group (Magiorakos Tables 1-5),
#    restricted to agents tested in the laboratory
# ----------------------------------------------------------------------------
S_AUREUS = {
    'Aminoglycosides': {'gentamicin'},
    'Anti-staphylococcal beta-lactams': {'oxacillin'},
    'Fluoroquinolones': {'ciprofloxacin'},
    'Folate pathway inhibitors': {'tmp-smx'},
    'Glycopeptides': {'vancomycin'},
    'Lincosamides': {'clindamycin'},
    'Macrolides': {'erythromycin'},
    'Oxazolidinones': {'linezolid'},
    'Phenicols': {'chloramphenicol'},
    'Tetracyclines': {'doxycycline', 'tetracycline'},
}
ENTEROCOCCUS = {
    # Aminoglycosides (high-level gentamicin): only routine gentamicin was
    # tested, so this category is not applied (see ENTEROCOCCAL_HIGH_LEVEL_GENTAMICIN).
    'Carbapenems': {'imipenem', 'meropenem'},
    'Fluoroquinolones': {'ciprofloxacin', 'levofloxacin'},
    'Glycopeptides': {'vancomycin'},
    'Oxazolidinones': {'linezolid'},
    'Penicillins': {'ampicillin'},
    'Tetracyclines': {'doxycycline', 'tetracycline'},
}
ENTEROBACTERALES = {
    'Aminoglycosides': {'amikacin', 'gentamicin'},
    'Antipseudomonal penicillins + beta-lactamase inhibitors': {'piperacillin-tazobactam',
                                                                'ticarcillin-clavulanate'},
    'Carbapenems': {'ertapenem', 'imipenem', 'meropenem'},
    'Non-extended-spectrum cephalosporins': {'cefazolin', 'cefuroxime', 'cephalexin',
                                             'cephalosporin i'},
    'Extended-spectrum cephalosporins': {'cefepime', 'cefotaxime', 'ceftazidime', 'ceftriaxone'},
    'Fluoroquinolones': {'ciprofloxacin'},
    'Folate pathway inhibitors': {'tmp-smx'},
    'Monobactams': {'aztreonam'},
    'Penicillins': {'amoxicillin', 'ampicillin'},
    'Penicillins + beta-lactamase inhibitors': {'amoxicillin-clavulanate', 'ampicillin-sulbactam'},
    'Phenicols': {'chloramphenicol'},
    'Polymyxins': {'colistin', 'polymyxin b'},
    'Tetracyclines': {'doxycycline', 'tetracycline'},
}
PSEUDOMONAS = {
    'Aminoglycosides': {'amikacin', 'gentamicin'},
    'Antipseudomonal carbapenems': {'imipenem', 'meropenem'},
    'Antipseudomonal cephalosporins': {'cefepime', 'ceftazidime'},
    'Antipseudomonal fluoroquinolones': {'ciprofloxacin', 'levofloxacin'},
    'Antipseudomonal penicillins + beta-lactamase inhibitors': {'piperacillin-tazobactam',
                                                                'ticarcillin-clavulanate'},
    'Monobactams': {'aztreonam'},
    'Polymyxins': {'colistin', 'polymyxin b'},
}
ACINETOBACTER = {
    'Aminoglycosides': {'amikacin', 'gentamicin'},
    'Antipseudomonal carbapenems': {'imipenem', 'meropenem'},
    'Antipseudomonal fluoroquinolones': {'ciprofloxacin', 'levofloxacin'},
    'Antipseudomonal penicillins + beta-lactamase inhibitors': {'piperacillin-tazobactam',
                                                                'ticarcillin-clavulanate'},
    'Extended-spectrum cephalosporins': {'cefepime', 'cefotaxime', 'ceftazidime', 'ceftriaxone'},
    'Folate pathway inhibitors': {'tmp-smx'},
    'Penicillins + beta-lactamase inhibitors': {'ampicillin-sulbactam'},
    'Polymyxins': {'colistin', 'polymyxin b'},
    'Tetracyclines': {'doxycycline', 'tetracycline'},
}

ENTEROBACTERALES_ORGS = {'Escherichia Coli', 'Klebsiella Pneumonia', 'Klebsiella spp',
                         'Enterobacter spp', 'Proteus spp', 'Providencia spp', 'Salmonella spp',
                         'Serratia spp', 'Morganella Morganii'}
PSEUDOMONAS_ORGS = {'Pseudonomas Aeruginosa', 'Pseudomonas spp'}
S_AUREUS_ORGS = {'Staphyloccoccus Aureus', 'Methicillin Resistant Staphylococcus Aureus'}

# Intrinsic resistance (CLSI M100): agents not assessed for these organisms
INTRINSIC = {
    'Klebsiella Pneumonia': {'amoxicillin', 'ampicillin', 'ticarcillin'},
    'Klebsiella spp': {'amoxicillin', 'ampicillin', 'ticarcillin'},
    'Enterobacter spp': {'amoxicillin', 'amoxicillin-clavulanate', 'ampicillin', 'ampicillin-sulbactam',
                         'cefazolin', 'cefoxitin', 'cefuroxime', 'cephalexin', 'cephalosporin i'},
    'Proteus spp': {'colistin', 'doxycycline', 'nitrofurantoin', 'polymyxin b', 'tetracycline',
                    'tigecycline'},
    'Providencia spp': {'amoxicillin', 'amoxicillin-clavulanate', 'ampicillin', 'cefazolin',
                        'cephalexin', 'cephalosporin i', 'colistin', 'doxycycline', 'nitrofurantoin',
                        'polymyxin b', 'tetracycline', 'tigecycline'},
    'Morganella Morganii': {'amoxicillin', 'amoxicillin-clavulanate', 'ampicillin', 'cefazolin',
                            'cefuroxime', 'cephalexin', 'cephalosporin i', 'colistin', 'doxycycline',
                            'nitrofurantoin', 'polymyxin b', 'tetracycline', 'tigecycline'},
    'Serratia spp': {'amoxicillin', 'amoxicillin-clavulanate', 'ampicillin', 'cefazolin', 'cefoxitin',
                     'cefuroxime', 'cephalexin', 'cephalosporin i', 'colistin', 'nitrofurantoin',
                     'polymyxin b'},
    'Acinobacter spp': {'amoxicillin', 'ampicillin', 'aztreonam'},
}


def organism_group(org):
    if org in ENTEROBACTERALES_ORGS:
        return 'Enterobacterales'
    if org in PSEUDOMONAS_ORGS:
        return 'Pseudomonas spp.'
    if org == 'Acinobacter spp':
        return 'Acinetobacter spp.'
    if org == 'Enterococcus spp':
        return 'Enterococcus spp.'
    if org in S_AUREUS_ORGS:
        return 'S. aureus'
    return None  # no Magiorakos definition


def categories(group, enterococcal_high_level_gentamicin=False, widen_fq=False):
    cats = {'Enterobacterales': ENTEROBACTERALES, 'Pseudomonas spp.': PSEUDOMONAS,
            'Acinetobacter spp.': ACINETOBACTER, 'Enterococcus spp.': ENTEROCOCCUS,
            'S. aureus': S_AUREUS}[group]
    cats = {k: set(v) for k, v in cats.items()}
    if group == 'Enterococcus spp.' and enterococcal_high_level_gentamicin:
        cats['Aminoglycosides (high-level gentamicin)'] = {'gentamicin'}
    if widen_fq:
        for k in cats:
            if 'luoroquinolone' in k:
                cats[k] |= {'ciprofloxacin', 'levofloxacin', 'ofloxacin', 'norfloxacin'}
    return cats


# ----------------------------------------------------------------------------
# 3. MDR classification
# ----------------------------------------------------------------------------
def classify(row, mrsa_definition='oxacillin', mrsa_rule=True,
             enterococcal_high_level_gentamicin=False, widen_fq=False,
             conflicting_as_resistant=True):
    """Return (status, categories resistant, categories tested).

    status: 'MDR', 'non-MDR', 'not classifiable' (not MDR and <3 categories
    tested) or 'not covered' (organism without a Magiorakos definition).
    """
    org = row[ORG]
    group = organism_group(org)
    if group is None:
        return 'not covered', np.nan, np.nan
    cats = categories(group, enterococcal_high_level_gentamicin, widen_fq)
    intrinsic = INTRINSIC.get(org, set())
    resistant = {a for a in row['resistant'] if a not in intrinsic}
    if not conflicting_as_resistant:
        resistant -= row['susceptible']
    tested = {a for a in (row['resistant'] | row['susceptible']) if a not in intrinsic}
    n_res = sum(1 for m in cats.values() if m & resistant)
    n_tested = sum(1 for m in cats.values() if m & tested)
    if group == 'S. aureus' and mrsa_rule:
        labelled = org == 'Methicillin Resistant Staphylococcus Aureus'
        is_mrsa = labelled if mrsa_definition == 'label' else ('oxacillin' in resistant)
        if is_mrsa:
            return 'MDR', n_res, n_tested
    if n_res >= 3:
        return 'MDR', n_res, n_tested
    if n_tested < 3:
        return 'not classifiable', n_res, n_tested
    return 'non-MDR', n_res, n_tested


PRIMARY = dict(mrsa_definition='oxacillin', mrsa_rule=True,
               enterococcal_high_level_gentamicin=False, widen_fq=False,
               conflicting_as_resistant=True)


def wilson(k, n, z=1.96):
    if n == 0:
        return (np.nan, np.nan)
    p = k / n
    d = 1 + z * z / n
    c = (p + z * z / (2 * n)) / d
    h = z * sqrt(p * (1 - p) / n + z * z / (4 * n * n)) / d
    return (100 * (c - h), 100 * (c + h))


def summarise(status):
    mdr = int((status == 'MDR').sum())
    covered = int((status != 'not covered').sum())
    classifiable = int(status.isin(['MDR', 'non-MDR']).sum())
    lo1, hi1 = wilson(mdr, covered)
    lo2, hi2 = wilson(mdr, classifiable)
    return dict(mdr=mdr, covered=covered, classifiable=classifiable,
                pct_covered=round(100 * mdr / covered, 1), ci_covered=(round(lo1, 1), round(hi1, 1)),
                pct_classifiable=round(100 * mdr / classifiable, 1),
                ci_classifiable=(round(lo2, 1), round(hi2, 1)))


# ----------------------------------------------------------------------------
# 4. Data
# ----------------------------------------------------------------------------
df = pd.read_excel(DATA, sheet_name='Raw data')
df['resistant'] = df.apply(lambda r: agents(r, RES_COLS), axis=1)
df['susceptible'] = df.apply(lambda r: agents(r, SUS_COLS), axis=1)
df['sampling_date'] = pd.to_datetime(df['Day of Sampling'], errors='coerce')
bact = df[df['Is bacterial']].copy().reset_index(drop=True)

res = bact.apply(lambda r: classify(r, **PRIMARY), axis=1, result_type='expand')
bact['mdr_status'], bact['categories_resistant'], bact['categories_tested'] = res[0], res[1], res[2]
bact['organism_group'] = bact[ORG].map(organism_group).fillna('not covered')

# possible colonisation or contamination
bact['possible_colonisation'] = (
    ((bact[ORG] == 'Coagulase-Negative Staphylococci (Cons)') & (bact['Type of Sample'] == 'Blood')) |
    bact['Type of Sample'].isin(['Sputum', 'Tracheal Aspirate', 'Throat Swab', 'Wound Swab']))
# sampled after the laboratory introduced VITEK (December 2024 onwards)
bact['vitek_period'] = bact['sampling_date'] >= '2024-12-01'

results = {}
results['primary'] = summarise(bact['mdr_status'])

# MDR by organism group
by_org = []
for g in ['Acinetobacter spp.', 'Enterobacterales', 'Enterococcus spp.', 'Pseudomonas spp.', 'S. aureus']:
    s = bact[bact.organism_group == g]
    st = s['mdr_status']
    covered = len(s)
    by_org.append(dict(group=g, mdr=int((st == 'MDR').sum()), non_mdr=int((st == 'non-MDR').sum()),
                       not_classifiable=int((st == 'not classifiable').sum()),
                       pct=round(100 * (st == 'MDR').sum() / covered, 1),
                       median_categories_tested=float(s['categories_tested'].median())))
results['by_organism'] = by_org
results['not_covered'] = bact[bact.mdr_status == 'not covered'][ORG].value_counts().to_dict()

# ----------------------------------------------------------------------------
# 5. Sensitivity analyses of the MDR estimate
# ----------------------------------------------------------------------------
variants = [
    ('Primary', PRIMARY),
    ('MRSA defined by laboratory label', dict(PRIMARY, mrsa_definition='label')),
    ('Enterococcal gentamicin read as high-level', dict(PRIMARY, enterococcal_high_level_gentamicin=True)),
    ('Fluoroquinolone category widened', dict(PRIMARY, widen_fq=True)),
    ('No automatic MRSA rule', dict(PRIMARY, mrsa_rule=False)),
    ('Agents recorded as both S and R counted as susceptible', dict(PRIMARY, conflicting_as_resistant=False)),
]
sens = []
for label, spec in variants:
    st = bact.apply(lambda r: classify(r, **spec)[0], axis=1)
    sens.append(dict(analysis=label, **summarise(st)))
for label, mask in [('Excluding possible colonisation/contamination', ~bact.possible_colonisation),
                    ('Excluding episodes sampled from December 2024 (VITEK period)', ~bact.vitek_period)]:
    sens.append(dict(analysis=label, **summarise(bact.loc[mask, 'mdr_status'])))
results['sensitivity'] = sens
results['conflicting_results_isolates'] = int(sum(1 for _, r in bact.iterrows()
                                                  if r['resistant'] & r['susceptible']))

# MDR among covered isolates with progressively more complete panels
completeness = []
for k in [3, 4, 5, 6]:
    s_k = bact[(bact.mdr_status != 'not covered') & (bact.categories_tested >= k)]
    m_k = int((s_k.mdr_status == 'MDR').sum())
    completeness.append(dict(min_categories_tested=k, mdr=m_k, n=len(s_k),
                             pct=round(100 * m_k / len(s_k), 1),
                             ci=tuple(round(v, 1) for v in wilson(m_k, len(s_k)))))
results['completeness'] = completeness

# ----------------------------------------------------------------------------
# 6. Factors associated with MDR (classifiable isolates)
# ----------------------------------------------------------------------------
INF4 = {'Urinary Tract Infection': 'UTI', 'Bloodstream Infection': 'BSI',
        'Skin And Soft Tissue Infection': 'SSTI', 'Respiratory Tract Infection': 'RTI/GI/other',
        'Gastro-Intestinal Infection': 'RTI/GI/other'}
INF5 = {'Urinary Tract Infection': 'UTI', 'Bloodstream Infection': 'BSI',
        'Skin And Soft Tissue Infection': 'SSTI', 'Respiratory Tract Infection': 'RTI',
        'Gastro-Intestinal Infection': 'GI/other'}


def prepare(d):
    d = d.copy().reset_index(drop=True)
    d['mdr'] = (d['mdr_status'] == 'MDR').astype(int)
    d['age_z'] = (d['Age'] - d['Age'].mean()) / d['Age'].std()
    d['female'] = (d['Gender'] == 'Female').astype(int)
    d['outpatient'] = (d['Care Details'] == 'Outpatient').astype(int)
    d['gram_positive'] = (d[GRAM] == 'Gram-Positive Cocci').astype(int)
    d['inf4'] = pd.Categorical(d['Type of Infection'].map(INF4),
                               categories=['UTI', 'BSI', 'SSTI', 'RTI/GI/other'])
    d['inf5'] = pd.Categorical(d['Type of Infection'].map(INF5),
                               categories=['UTI', 'BSI', 'SSTI', 'RTI', 'GI/other'])
    d['patient'] = d['Participant Unique ID']
    d['year'] = d['sampling_date'].dt.year
    return d


COV = 'age_z + female + outpatient + gram_positive'
F4 = 'mdr ~ ' + COV + ' + C(inf4, Treatment("UTI"))'
F5 = 'mdr ~ ' + COV + ' + C(inf5, Treatment("UTI"))'


def poisson(formula, d):
    return smf.glm(formula, d, family=sm.families.Poisson()).fit(cov_type='HC0')


def firth_logistic(formula, d, max_iter=100, tol=1e-8):
    """Logistic regression with Firth's penalised likelihood."""
    y, X = patsy.dmatrices(formula, d, return_type='dataframe')
    names = X.columns
    y = y.values.ravel()
    X = X.values
    beta = np.zeros(X.shape[1])
    for _ in range(max_iter):
        eta = X @ beta
        p = 1 / (1 + np.exp(-eta))
        W = p * (1 - p)
        XtWX = X.T @ (X * W[:, None])
        inv = np.linalg.inv(XtWX)
        h = np.einsum('ij,jk,ik->i', X * np.sqrt(W)[:, None], inv, X * np.sqrt(W)[:, None])
        U = X.T @ (y - p + h * (0.5 - p))
        step = inv @ U
        beta += step
        if np.max(np.abs(step)) < tol:
            break
    se = np.sqrt(np.diag(inv))
    z = beta / se
    pv = 2 * stats.norm.sf(np.abs(z))
    return pd.DataFrame({'coef': beta, 'se': se, 'p': pv}, index=names)


def extract(fit, label, exponentiate=True):
    out = {'model': label, 'n': int(fit.nobs) if hasattr(fit, 'nobs') else None}
    ci = fit.conf_int()
    for k in fit.params.index:
        if k == 'Intercept':
            continue
        e = np.exp if exponentiate else (lambda v: v)
        out[k] = (round(float(e(fit.params[k])), 2), round(float(e(ci.loc[k][0])), 2),
                  round(float(e(ci.loc[k][1])), 2), round(float(fit.pvalues[k]), 3))
    return out


def extract_firth(tab, label, n):
    out = {'model': label, 'n': n}
    for k in tab.index:
        if k == 'Intercept':
            continue
        b, se, p = tab.loc[k, 'coef'], tab.loc[k, 'se'], tab.loc[k, 'p']
        out[k] = (round(float(np.exp(b)), 2), round(float(np.exp(b - 1.96 * se)), 2),
                  round(float(np.exp(b + 1.96 * se)), 2), round(float(p), 3))
    return out


cls = prepare(bact[bact.mdr_status.isin(['MDR', 'non-MDR'])])
cov_all = prepare(bact[bact.mdr_status != 'not covered'])

models = []
models.append(extract(poisson(F4, cls), 'Primary (modified Poisson, classifiable isolates)'))
models.append(extract(poisson(F5, cls), 'Five infection groups'))
models.append(extract(poisson(F4, cov_all), 'All covered isolates (not classifiable counted as non-MDR)'))
gee = smf.gee(F4, groups='patient', data=cls, family=sm.families.Poisson(),
              cov_struct=sm.cov_struct.Exchangeable()).fit()
models.append(extract(gee, 'GEE, patient as cluster (exchangeable)'))
first = cls[cls['First bacterial episode per patient']].reset_index(drop=True)
models.append(extract(poisson(F4, first), 'First bacterial episode per patient'))
nocol = cls[~cls.possible_colonisation].reset_index(drop=True)
models.append(extract(poisson(F4, nocol), 'Excluding possible colonisation/contamination'))
noent = cls[cls.organism_group != 'Enterococcus spp.'].reset_index(drop=True)
models.append(extract(poisson(F4, noent), 'Excluding Enterococcus spp.'))
novitek = cls[~cls.vitek_period].reset_index(drop=True)
models.append(extract(poisson(F4, novitek), 'Excluding episodes sampled from December 2024'))
models.append(extract(smf.glm(F4, cls, family=sm.families.Binomial()).fit(), 'Logistic regression (aOR)'))
models.append(extract_firth(firth_logistic(F4, cls), 'Firth penalised logistic regression (aOR)', len(cls)))
models.append(extract(smf.glm(F5, cls, family=sm.families.Binomial()).fit(),
                      'Logistic regression, five infection groups (aOR)'))
models.append(extract_firth(firth_logistic(F5, cls), 'Firth penalised logistic regression, five infection groups (aOR)',
                            len(cls)))
results['models'] = models
results['n'] = dict(classifiable=len(cls), first_episode=len(first), no_colonisation=len(nocol),
                    no_enterococcus=len(noent), covered=len(cov_all), no_vitek=len(novitek))

# calendar-year trend (unadjusted modified Poisson model on year of sampling)
trend = smf.glm('mdr ~ year', cls.dropna(subset=['year']), family=sm.families.Poisson()).fit(cov_type='HC0')
ci = trend.conf_int().loc['year']
results['year_trend'] = (round(float(np.exp(trend.params['year'])), 2), round(float(np.exp(ci[0])), 2),
                         round(float(np.exp(ci[1])), 2), round(float(trend.pvalues['year']), 2))

# cancer stage, complete-case model
cc = cls.dropna(subset=['Stage of Cancer']).copy().reset_index(drop=True)
# stage I contains only three isolates, none MDR; stages I and II are combined
cc['stage_grp'] = cc['Stage of Cancer'].replace({'I': 'I-II', 'II': 'I-II'})
with_stage = poisson(F4 + ' + C(stage_grp)', cc)
# joint robust (HC0) Wald test of the stage terms
idx = [i for i, k in enumerate(with_stage.params.index) if 'stage_grp' in k]
b = with_stage.params.values[idx]
V = with_stage.cov_params().values[np.ix_(idx, idx)]
wald = float(b @ np.linalg.inv(V) @ b)
results['stage'] = dict(missing=int(cls['Stage of Cancer'].isna().sum()), complete_case_n=len(cc),
                        joint_p=round(float(stats.chi2.sf(wald, len(idx))), 2),
                        test='robust Wald, stage I-II combined')

# ----------------------------------------------------------------------------
# 7. Table 3: MDR versus non-MDR among classifiable isolates
# ----------------------------------------------------------------------------
mdr, non = cls[cls.mdr == 1], cls[cls.mdr == 0]


def chi_p(col):
    t = pd.crosstab(cls[col], cls['mdr'])
    return float(stats.chi2_contingency(t)[1])  # Yates' correction applied to 2x2 tables


def counts(col, levels):
    rows = []
    for lv in levels:
        a = int((mdr[col] == lv).sum())
        b = int((non[col] == lv).sum())
        rows.append((lv, a, round(100 * a / len(mdr), 1), b, round(100 * b / len(non), 1)))
    return rows


q = lambda s: (float(s.median()), float(s.quantile(.25)), float(s.quantile(.75)))
table3 = {
    'n_mdr': len(mdr), 'n_non': len(non), 'age_sd': round(float(cls.Age.std()), 1),
    'age': dict(mdr=q(mdr.Age), non=q(non.Age),
                p=round(float(stats.mannwhitneyu(mdr.Age, non.Age, alternative='two-sided').pvalue), 3)),
    'sex': dict(rows=counts('Gender', ['Female', 'Male']), p=round(chi_p('Gender'), 3)),
    'setting': dict(rows=counts('Care Details', ['Outpatient', 'Inpatient']), p=round(chi_p('Care Details'), 3)),
    'stage': dict(rows=[(lv, int((mdr['Stage of Cancer'] == lv).sum()),
                         round(100 * (mdr['Stage of Cancer'] == lv).sum() / mdr['Stage of Cancer'].notna().sum(), 1),
                         int((non['Stage of Cancer'] == lv).sum()),
                         round(100 * (non['Stage of Cancer'] == lv).sum() / non['Stage of Cancer'].notna().sum(), 1))
                        for lv in ['I', 'II', 'III', 'IV']],
                  p=round(float(stats.chi2_contingency(pd.crosstab(cc['Stage of Cancer'], cc['mdr']))[1]), 3)),
    'infection': dict(rows=counts('inf4', ['UTI', 'BSI', 'SSTI', 'RTI/GI/other']), p=round(chi_p('inf4'), 3)),
    'infection_rti_n': int((cls['inf5'] == 'RTI').sum()),
    'infection_gi_n': int((cls['inf5'] == 'GI/other').sum()),
    'gram': dict(rows=counts('gram_positive', [1, 0]), p=float(chi_p('gram_positive'))),
}
results['table3'] = table3
results['mdr_uti_of_all_mdr'] = (int(((bact.mdr_status == 'MDR') &
                                      (bact['Type of Infection'] == 'Urinary Tract Infection')).sum()),
                                 int((bact.mdr_status == 'MDR').sum()))
results['not_classifiable_covered'] = int((bact.mdr_status == 'not classifiable').sum())
results['vitek_period_episodes'] = int(bact.vitek_period.sum())

# ----------------------------------------------------------------------------
# 8. Output
# ----------------------------------------------------------------------------
with open(os.path.join(OUT, 'results.json'), 'w') as f:
    json.dump(results, f, indent=1, default=str)
cols = [ORG, 'Type of Infection', 'organism_group', 'categories_resistant', 'categories_tested', 'mdr_status',
        'possible_colonisation', 'vitek_period']
bact[['Participant Unique ID'] + cols].to_csv(os.path.join(OUT, 'mdr_classification_by_isolate.csv'), index=False)

p = results['primary']
print('Bacterial isolates %d; covered by Magiorakos definitions %d' % (len(bact), p['covered']))
print('MDR %d/%d = %.1f%% (95%% CI %.1f-%.1f)' % (p['mdr'], p['covered'], p['pct_covered'], *p['ci_covered']))
print('MDR among classifiable %d/%d = %.1f%% (95%% CI %.1f-%.1f)'
      % (p['mdr'], p['classifiable'], p['pct_classifiable'], *p['ci_classifiable']))
print('Results written to', OUT)
```

# **S5. Antimicrobial resistance among WHO priority pathogens**

**Table S5. Antimicrobial resistance among WHO priority pathogens in cancer patients with culture-confirmed infection, King Faisal Hospital, Kigali, Rwanda, 2019–2024.**

| **WHO priority category** | **Pathogen–resistance phenotype** | **Isolates in cohort (n)** | **Isolates tested (n)** | **Resistant (n)** | **Resistance (%)** | **95% CI (%)** |
| --- | --- | --- | --- | --- | --- | --- |
| Critical | Third-generation cephalosporin-resistant Enterobacterales | 160 | 142 | 99 | 69.7 | 62–77 |
| Critical | Carbapenem-resistant Enterobacterales | 160 | 148 | 12 | 8.1 | 5–14 |
| Critical | Carbapenem-resistant *Acinetobacter* spp. | 13 | 13 | 8 | 61.5 | 36–82 |
| Critical | Carbapenem-resistant *Pseudomonas* spp. | 23 | 22 | 7 | 31.8 | 16–53 |
| High | Methicillin-resistant *Staphylococcus aureus* (oxacillin-resistant) | 11 | 10 | 4 | 40.0 | 17–69 |
| High | Vancomycin-resistant *Enterococcus* spp. | 25 | 20 | 0 | 0.0 | 0–16 |
| High | Fluoroquinolone-resistant *Salmonella* spp. | 1 | 1 | 0 | 0.0 | 0–79 |
| Medium | Penicillin-resistant *Streptococcus* spp. | 11 | 0 | – | not tested | – |

Denominators are the isolates of the relevant organism group tested against at least one agent in the class; they differ between rows because susceptibility panels were not uniform. Enterobacterales comprise *E. coli* (n = 101), *K. pneumoniae* (n = 24), other *Klebsiella* spp. (n = 8), *Proteus* spp. (n = 10), *Enterobacter* spp. (n = 8), *Providencia* spp. (n = 4), *Serratia* spp. (n = 2), *Morganella morganii* (n = 2) and *Salmonella* spp. (n = 1). *Pseudomonas* pools *P. aeruginosa* (n = 19) and non-*aeruginosa* species (n = 4). Methicillin resistance is defined phenotypically by oxacillin resistance. Confidence intervals are Wilson score intervals; estimates from fewer than 20 tested isolates should be regarded as preliminary.

# **S6. Empirical antibiotic agents by WHO AWaRe category**

**Table S6.1. Empirical antibiotic agents by WHO AWaRe category among the 105 infection episodes with therapy documented in the electronic medical record.**

| **WHO AWaRe category** | **Antibiotic agent** | **Episodes in which prescribed (n)** | **% of 105 documented episodes** |
| --- | --- | --- | --- |
| Watch | Ceftriaxone | 54 | 51.4 |
| Watch | Meropenem | 14 | 13.3 |
| Watch | Vancomycin | 6 | 5.7 |
| Watch | Ciprofloxacin | 6 | 5.7 |
| Watch | Levofloxacin | 4 | 3.8 |
| Watch | Erythromycin | 4 | 3.8 |
| Watch | Piperacillin–tazobactam | 2 | 1.9 |
| Watch | Cefuroxime | 2 | 1.9 |
| Watch | Piperacillin | 1 | 1.0 |
| Watch | Cefixime | 1 | 1.0 |
| Watch | Azithromycin | 1 | 1.0 |
| Access | Metronidazole | 12 | 11.4 |
| Access | Amoxicillin–clavulanate | 8 | 7.6 |
| Access | Cloxacillin | 3 | 2.9 |
| Access | Trimethoprim–sulfamethoxazole | 3 | 2.9 |
| Access | Doxycycline | 1 | 1.0 |
| Access | Flucloxacillin | 1 | 1.0 |
| Access | Ampicillin | 1 | 1.0 |
| Reserve | Polymyxin B | 1 | 1.0 |

**Table S6.2. Episodes containing at least one agent of each category**

| **WHO AWaRe category** | **Episodes (n)** | **% of 105 documented episodes** |
| --- | --- | --- |
| Watch | 88 | 83.8 |
| Access | 27 | 25.7 |
| Reserve | 1 | 1.0 |

Counts are episodes in which the agent formed part of the documented empirical regimen. 16 of the 105 regimens contained more than one agent, so the agent counts sum to more than 105 and the category rows overlap; percentages are of the 105 documented episodes throughout. 19 distinct agents were prescribed. Because 100 of the 105 documented regimens were prescribed to inpatients, these data describe predominantly inpatient practice.
